# Oral neonatal antibiotic treatment perturbs gut microbiota and aggravates central nervous system autoimmunity in Dark Agouti rats

**DOI:** 10.1038/s41598-018-37505-7

**Published:** 2019-01-29

**Authors:** Suzana Stanisavljević, Aleksa Čepić, Svetlana Bojić, Katarina Veljović, Sanja Mihajlović, Neda Đedović, Bojan Jevtić, Miljana Momčilović, Milica Lazarević, Marija Mostarica Stojković, Đorđe Miljković, Nataša Golić

**Affiliations:** 1 Department of Immunology, Institute for Biological Research “Siniša Stanković”, University of Belgrade, Belgrade, Serbia; 2 Laboratory for Molecular Microbiology, Institute of Molecular Genetics and Genetic Engineering, University of Belgrade, Belgrade, Serbia; 3 HITTest doo, Belgrade, Serbia; 4 Institute for Microbiology and Immunology, School of Medicine, University of Belgrade, Belgrade, Serbia

## Abstract

Gut microbiota dysbiosis has been considered the essential element in the pathogenesis of multiple sclerosis and its animal model, experimental autoimmune encephalomyelitis (EAE). Antibiotics were administered orally to Dark Agouti (DA) rats early in their life with the aim of perturbing gut microbiota and investigating the effects of such intervention on the course of EAE. As a result, the diversity of the gut microbiota was reduced under the influence of antibiotics. Mainly, *Firmicutes* and *Actinobacteria* were replaced by *Proteobacteria* and *Bacteroidetes*, while decreased proportions of *Clostridia* and *Bacilli* classes were accompanied by an increase in Gamma*-Proteobacteria* in antibiotic-treated animals. Interestingly, a notable decrease in the *Helicobacteraceae*, *Spirochaetaceae* and *Turicibacteriaceae* was scored in antibiotic-treated groups. Also, levels of short chain fatty acids were reduced in the faeces of antibiotic-treated rats. Consequently, aggravation of EAE, paralleled with stronger immune response in lymph nodes draining the site of immunization, and increased inflammation within the CNS, were observed in antibiotic-treated DA rats. Thus, the alteration of gut microbiota leads to an escalation of CNS-directed autoimmunity in DA rats. The results of this study indicate that antibiotic use in early life may have subsequent unfavourable effects on the regulation of the immune system.

## Introduction

The intestinal microbiota has become increasingly appreciated as an important, if not the major external factor of immune response regulation^1,2^. A stable gut microbiota which is in balance with gut-associated lymphoid tissue (GALT) is a prerequisite for immune homeostasis^1^. The ratio between effector T helper (Th) cells and regulatory T (Treg) cells determines the inflammatory vs. regulatory milieu in the GALT, while the intestinal microbiota has a profound influence on Th/Treg balance^3^. Disturbance of the equilibrium predisposes one to various diseases^3^, including autoimmunity directed against the central nervous system (CNS), as observed in multiple sclerosis and its animal model, experimental autoimmune encephalomyelitis (EAE)^4^. It has been also shown that encephalitogenic cells migrating into the GALT can be restrained there, expelled into gut lumen and even transformed into Treg which are effective against CNS autoimmunity^5,6^. A balanced intestinal microbiota stimulates a regulatory milieu in the GALT through the production and release of various immunomodulatory compounds, including short chain fatty acids (SCFA) and polysaccharide A^3^. Thus, a stable and balanced microbiota seems to be a prerequisite for the prevention of various immune-mediated disorders.

Gut microbiota dysbiosis has been observed in various autoimmune disorders, including multiple sclerosis, where changes in gut microbiota composition in comparison to healthy subjects have been detected^7–10^. Consequently, gut microbiota dysbiosis has been suggested as an important element of multiple sclerosis pathogenesis^4,11^. Although differences in gut microbiota composition between multiple sclerosis patients and healthy subjects are evident, it is still not clear if that dysbiosis is a predisposing factor for multiple sclerosis or a consequence of the disease^4,11^. However, it has recently been shown that transfer of multiple sclerosis patients’ intestinal microbiota potentiates Th1/Th17 over Treg, and enhances spontaneous and active EAE in transgenic and C57Bl/6 mice, respectively^12,13^.

Although antibiotics are generally considered safe medications with mild and rare side effects, there is an increasing awareness of serious consequences of antibiotic use on gut microbiota^14^. Indeed, a rising number of observational, clinical, and epidemiologic studies focused on children and antibiotics use show that antibiotic exposure-related dysbiosis of intestinal microbiota increases the risks for various diseases such as obesity, diabetes, inflammatory bowel diseases, celiac disease, allergies and asthma^14^. Recent studies have shown that global antibiotics use increased in the first decade of the 21^st^ century^15,16^. Importantly, antibiotics are the most commonly prescribed paediatric drugs, taking a share of more than 30% of all drugs prescribed to children younger than two years^17^. Specifically, the shares of amoxicillin and azithromycin were 17.1% and 6.3% in year 2010, respectively. Therefore, studies that explore the autoimmunity-related effects of antibiotics on intestinal microbiota are valuable for assessing the potential risks of antibiotic use.

Here, EAE-susceptible Dark Agouti (DA) rats were treated with antibiotics early in their lifetime and, as a consequence, their gut microbiota was disturbed and production of SCFA reduced. When challenged with encephalitogenic immunization later in their lives, at the time when gut microbiota dysbiosis was no longer evident, the rats that had been treated with antibiotics had more severe EAE in comparison to their untreated counterparts. The increased severity of EAE was paralleled with increased Th1 and Th17 activity and decreased Treg in immune compartments and within the CNS.

## Results

### Effects of antibiotic treatment on EAE

**Figure 1:**
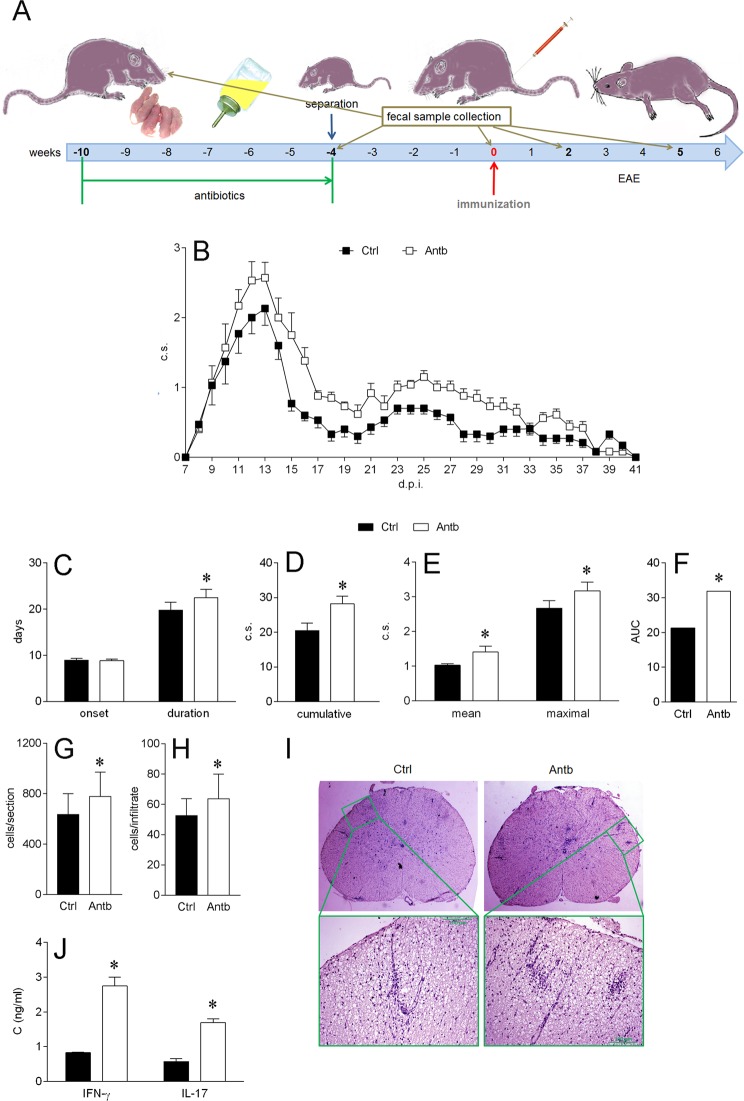
Antibiotics aggravate EAE in DA rats. DA rats were untreated (Ctrl) or treated with antibiotics (Antb) and immunized with SCH + CFA as depicted in the scheme (**A**). Clinical score was determined daily and various EAE parameters were determined (**B**–**H**). Representative micrographs of spinal cord sections are presented (**I**). Cytokine levels were determined in spinal cord homogenates obtained on 14 d.p.i. (**J**). Data are presented as mean +/− SD from 15 rats per group (**B**–**F**), from 17 sections per group (**G**,**H**) or from 2 rats per group (**J**). *p < 0.05, Antb vs. Ctrl.

Gravid DA rats were treated with antibiotics in drinking water starting two weeks before giving birth. The treatment continued for an additional four weeks after birth (Fig. 1A). Thus, the infants were exposed to antibiotics transferred to milk during nursing and directly through drinking water. EAE immunization was performed on 8-week-old rats. DA rats treated with antibiotics experienced more severe EAE than control rats (Fig. 1B), as evidenced by longer duration of the disease (Fig. 1C), higher cumulative clinical score (c.s.) mean (Fig. 1D), and maximal c.s. (Fig. 1E), higher area under the curve (Fig. 1F), more infiltrating immune cells per spinal cord section (Fig. 1G) and more cells per infiltrate (Fig. 1H). Also, higher levels of IFN-γ and IL-17 were detected in the spinal cords of antibiotic-treated DA rats (Fig. 1I).

### Effects of antibiotic treatment on gut microbiota

**Figure 2:**
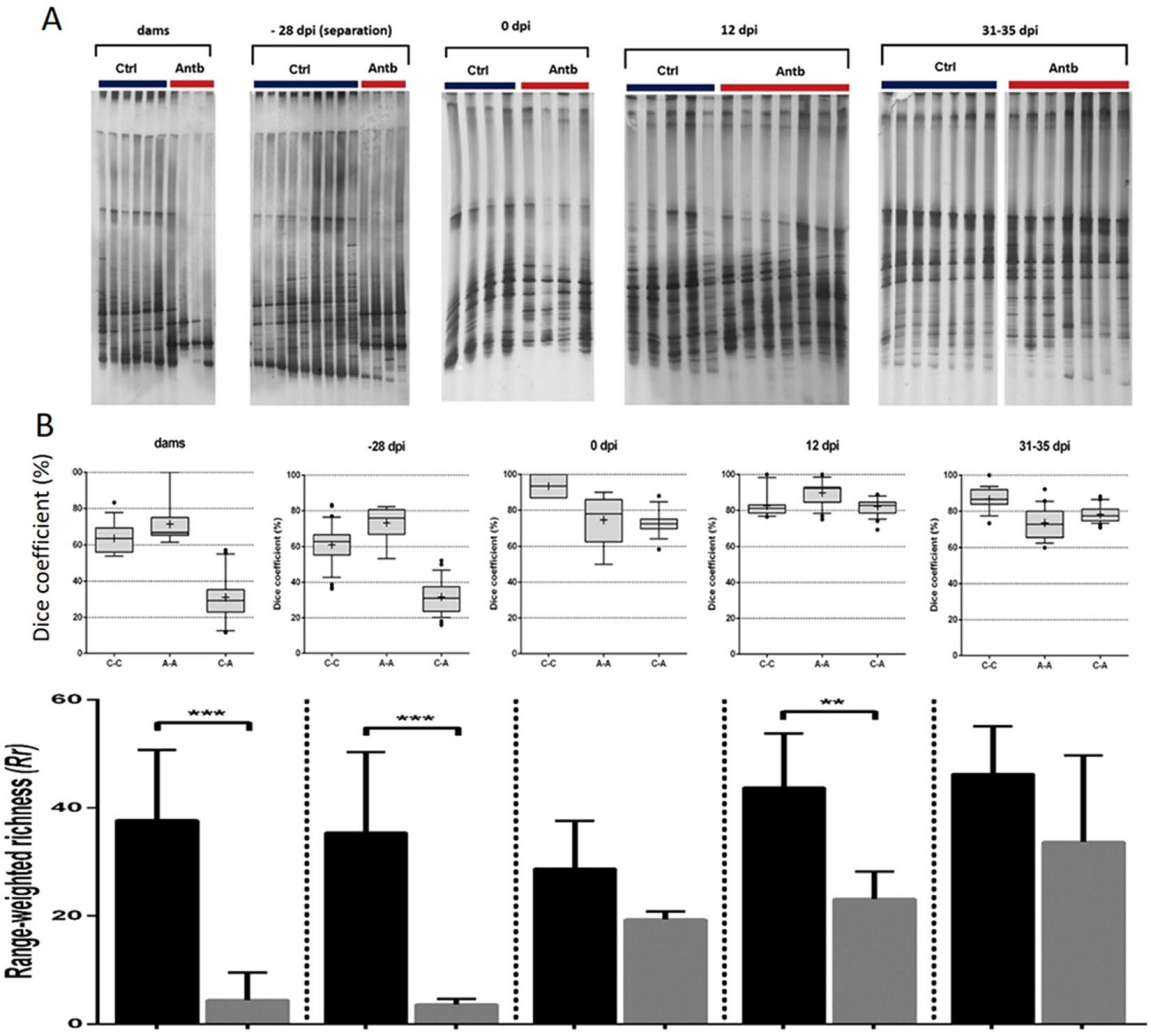
Effects of antibiotic treatment on DA rat gut microbiota composition. (**A**) Denaturing gradient gel electrophoresis (DGGE) profiles of rDNA amplicons obtained using universal primers (U968-GC and L1401) and DNA isolated from the faecal samples of DA rats. Each column represents a DGGE pattern of one rat: Ctrl – control animals; Antb – antibiotic-treated rats; dams – time of delivery (7–14 days after the treatment with antibiotic was started); −28 d.p.i. - offspring at the time of separation (28 days before the immunization,); 0 d.p.i. – immunization (SCH + CFA); 12 d.p.i. - EAE peak; 31–35 d.p.i. – recovery. (**B**) Box-plot diagrams of DGGE profiles’ similarity levels based on Dice similarity coefficient. C-C similarity among samples in the control group; A-A similarity among samples in the antibiotic-treated group; C-A similarity between samples of the control and antibiotic-treated group. (**C**) Diversity estimated on the basis of the *Range-weighted richness* - Rr from the feces of rats of control and antibiotic-treated rats. Values are presented as arithmetic mean ± SD *p < 0.05, **p < 0.01, ***p < 0.001.

In order to determine the influence of antibiotic treatment on gut microbiota, DGGE analyses of rDNA amplicons obtained by using DNA isolated from faecal samples as templates, and universal primers complementary to 16S rRNA regions of Eubacteria, respectively, were used (Fig. 2A). The lowest similarity between the antibiotic-treated and control groups was scored in dams and in offspring at the time of separation (31.0 ± 2.6% and 31.7 ± 1.6%, respectively), indicating the greatest influence of antibiotic treatment on microbiota diversity in the groups. The highest similarity between antibiotic-treated and control groups was scored after the EAE induction (72.4 ± 1.7% at 0 d.p.i., 82.2 ± 0.8% at 12 d.p.i. and 78.2 ± 0.7% at 31–35 d.p.i.), indicating a recovery of microbiota four weeks after the completion of antibiotic therapy (Fig. 2B). Antibiotic treatment led to a significant decrease in bacterial diversity in dams (p<0.001) and offspring at the time of separation (p < 0.001), and at the peak of the disease (p<0.01), while no significant differences were observed at other time points (Fig. 2C).

On the other hand, a high similarity of DGGE profiles within the groups, both antibiotic-treated and control rats, was measured, ranging from 61.0 ± 1.9% to 93.5 ± 2.9% within the control groups and from 71.4 ± 5.8% to 89.8 ± 1.4% within the antibiotic-treated groups. Therefore, 16S rRNA metagenomic sequencing was performed on one randomly chosen faecal sample from each group in order to determine the main microbial players responsible for aggravation of EAE symptoms in DA rats.

**Figure 3:**
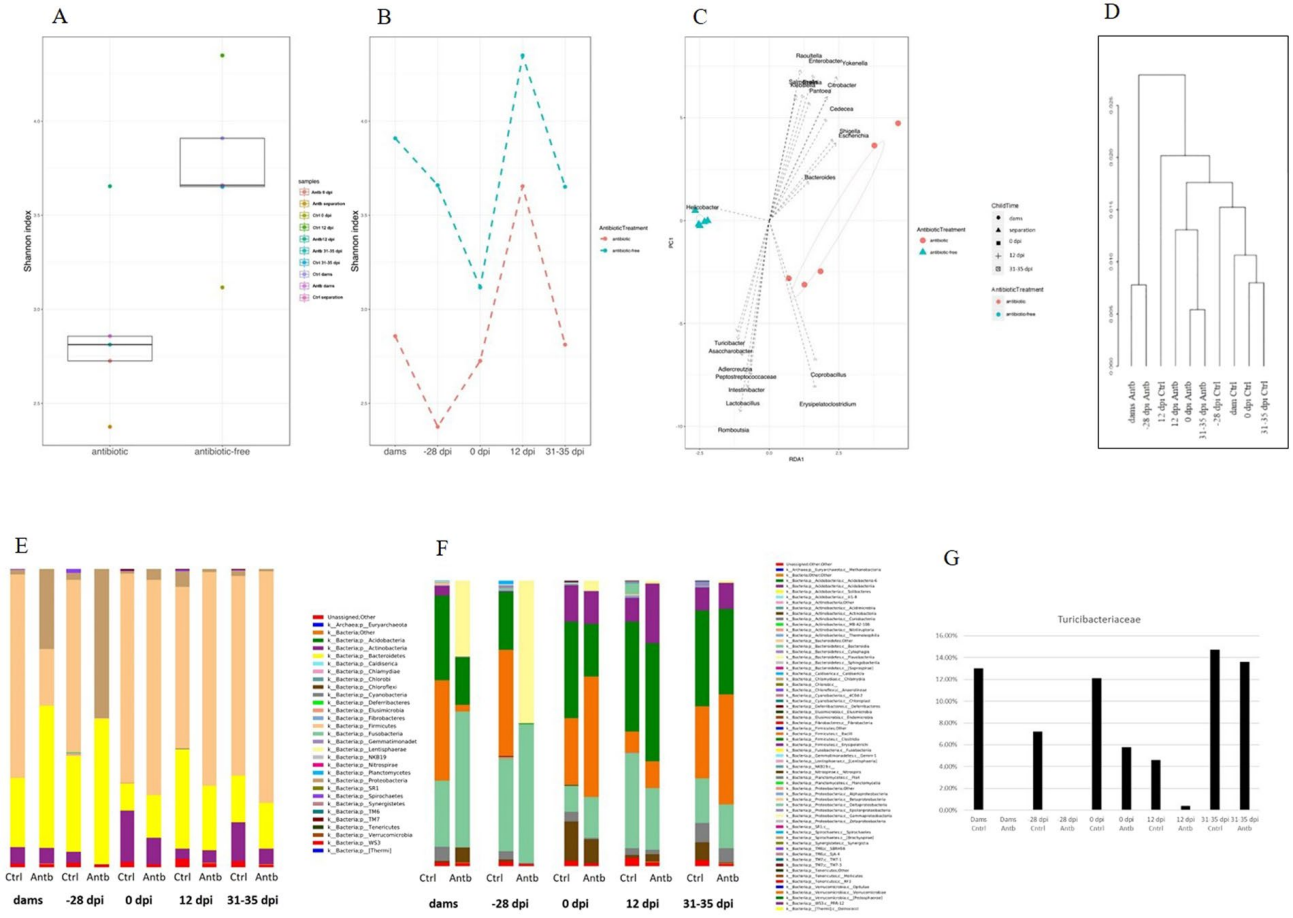
Antibiotics effects on gut microbiota. Samples are obtained from dams – time of delivery (7–14 days after the treatment with antibiotic was started); −28 d.p.i. - offspring at the time of separation (28 days before the immunization,); 0 d.p.i. – immunization (SCH + CFA); 12 d.p.i. - EAE peak; 31–35 d.p.i. – recovery. (**A**) alpha-diversity plot calculated by Shannon and Simpson between all antibiotic-treated (Antb) and untreated (Ctrl) DA rats alpha diversity, all samples resampled to have equal library size, grouped by antibiotic treatment; (**B**) alpha-diversity calculated by Shenon and Simpson between individual antibiotic-treated (antibiotic) and untreated (antibiotic free) rats’ groups, all samples resampled to have equal library size, grouped by antibiotic treatment. (**C**) Redundancy analysis (RDA) plot, genus level, constrained on antibiotic usage; (**D**) Hierarchical clustering, weighted unifrac analysis; (**E**) relative abundances at the phylum level; (**F**) relative abundances at the class level; (**G**) relative abundance of *Turicibacteriaceae*.

The Shannon diversity analysis of 16S rRNA gene metagenomics sequencing revealed differences in alpha-diversity between control and antibiotic-treated groups. In accordance with results obtained by DGGE, alpha-diversity was lower in antibiotic-treated groups compared to untreated controls (Fig. 3A). Interestingly, an increase in alpha diversity was observed at the time of EAE peak, in both antibiotic-treated and untreated groups (Fig. 3B). Redundancy analysis (RDA) at the genus level, constrained to antibiotic usage, revealed the highest effect of antibiotic use on *Hellicobacter*. On the other hand, in antibiotic-treated dams higher numbers of *Proteobacteria* (particularly of the *Esherichia* and *Shigela* genera) and *Bacteroidetes* (*Bacteroides* sp.) was observed (Fig. 3C). In addition, samples from antibiotic-treated and control rats were grouped into separate clades regarding their gut microbiota composition, where the antibiotic-treated dams and offspring at the time of separation were clustered together. An exemption was observed in the control sample at the time of EAE peak, clustered with antibiotic rat samples (Fig. 3D).

Analyses of the relative abundance of detected bacteria within each sample at a phylum level revealed the highest dysbiosis of microbiota composition caused by antibiotic treatment in antibiotic-treated dams and offspring at the time of separation, where *Firmicutes* and *Actinobacteria* were entirely replaced by *Proteobacteria* and *Bacteroidetes*. Interestingly, the control samples from rats at the time of separation and at EAE peak had a higher proportion of *Bacteroidetes* comparing to other control samples, while the highest proportion of *Actinobacteria* was scored in samples from control rats at the time of EAE induction (Fig. 3E). In addition, at the class level, the analysis of relative abundance of detected bacteria within each sample showed specific changes among the *Firmicute* phylum. Namely, a decreased proportion of *Clostridia* and particularly a decrease in the *Bacilli* class were observed in antibiotic-treated dams. Moreover, this analysis revealed that the *Proteobacteria* replacing *Firmucutes* in antibiotic-treated offspring (−28 d.p.i.) were mainly Gamma*-Proteobacteria* (Fig. 3F).

Finally, at the family level the most notable decrease was in the *Helicobacteraceae* and *Spirochaetaceae* (p. adjusted = 0.0942 and 0.083, respectively) in the antibiotic-treated group, as well as an increase in *Bacteroidaceae* and *Enterobacteriaceae* families (p. adjusted = 0.0831 and 0.083 respectively). Interestingly, a decrease in the abundance of *Turicibacteriaceae* in antibiotic-treated rats compared to control groups was scored in all groups from dams to the peak of disease. The abundance of *Turicibacteriaceae* was restored to the control level only from 31–35 days after immunization (Fig. 3G). On the other hand, at the genus level the most prominent changes were noted in *Ruminococcus*, *Bacteroides*, CF231, *Enterobacter*, *Treponema* and YRC22 genera (p adjusted = 0.0804, 0.0812, 0.0812, 0.0812, 0.0812 and 0.094 respectively).

### Effects of antibiotic treatment on SCFA

**Figure 4:**
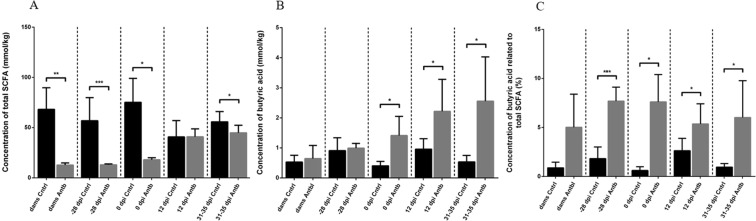
Effect of antibiotic treatment on faecal concentration of short-chain fatty acids (SCFA). Samples are obtained from dams – time of delivery (7–14 days after the treatment with antibiotic was started); −28 d.p.i. - offspring at the time of separation (28 days before the immunization,); 0 d.p.i. – immunization (SCH + CFA); 12 d.p.i. - EAE peak; 31–35 d.p.i. – recovery. (**A**) Concentration of total SCFA from feces of antibiotic-treated and untreated rats. (**B**) Concentration of butyric acid from the feces of antibiotic-treated and untreated rats. (**C**) Concentration of butyric acid versus total SCFA from feces of antibiotic-treated and untreated rats. Values are presented as arithmetic mean ± SD; *p < 0.05, **p < 0.01, ***p < 0.001.

Total SCFA concentrations in faecal samples of antibiotic-treated and untreated DA rats were analysed. The results revealed significantly lower amounts of SCFA detected in antibiotic-treated rats, except at the time of EAE peak (Fig. 4A). Interestingly, the concentration of butyric acid was significantly higher in antibiotic-treated rats than in the control animals on the day of immunization (p < 0.05), 12 days after immunization (12 d.p.i.) (p < 0.05) and 31–35 days after the immunization (p < 0.05) (Fig. 4B). Similarly, the relative abundance of butyric acid in total SCFA was significantly higher in the antibiotic-treated offspring, but not in the dams (Fig. 4C).

### Effects of antibiotic treatment on lymph node cells

**Figure 5:**
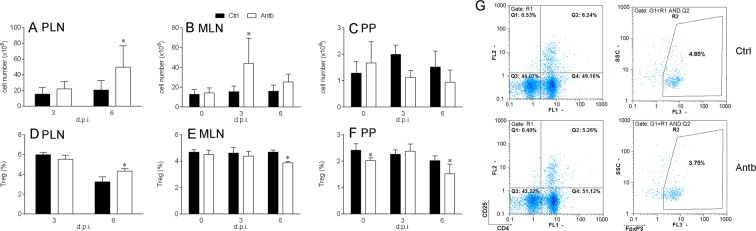
Antibiotics effects on lymph nodes. DA rats were untreated (Ctrl) or treated with antibiotics (Antb). Rats were immunized with SCH + CFA. Cellularity of PLN (**A**), MLN (**B**) and PP (**C**) was determined. Proportion of Treg among cells of PLN (**D**), MLN (**E**) and PP (**F**) was determined. Cells were gated on live cells (R1) and CD4^+^CD25^+^ cells (Q2). Representative dot plots of MLN cells obtained on 6 d.p.i. showing gating strategy for Treg are presented (**G**). Data are presented as mean +/− SD from from 6 rats per group (**A**–**F**). *p < 0.05, Antb vs. Ctrl.

Lymph nodes draining the site of injection were isolated on 3 d.p.i. and 6 d.p.i., while MLN and PP were isolated before the immunization (0 d.p.i.) and at 3 d.p.i. and 6 d.p.i. Increased cellularity was observed in PLN at 6 d.p.i. and in MLN on 3 d.p.i. in antibiotic-treated rats (Fig. 5A,B), while cellularity of PP remained stable irrespective of the treatment (Fig. 5C). Proportion of Treg was increased among PLN, but decreased among MLN and PP at 6 d.p.i. in antibiotic-treated DA rats (Fig. 5D–G).

**Figure 6:**
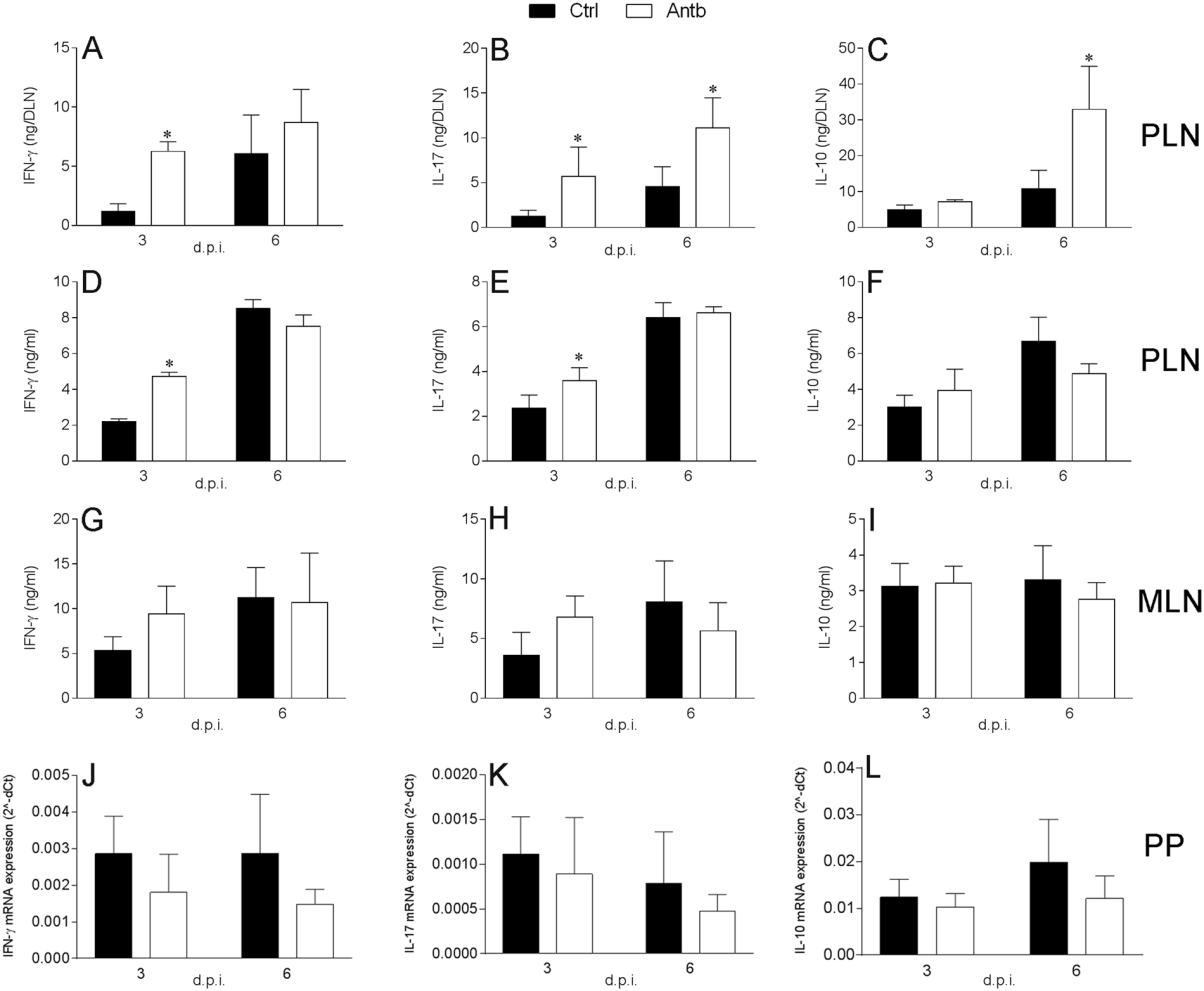
Cytokine production in antibiotic-treated DA rats. DA rats were untreated (Ctrl) or treated with antibiotics Antb. Rats were immunized with MBP + CFA for PLN and with SCH + CFA for MLN and PP. Spontaneous release of cytokines per PLN (**A**–**C**), MBP-stimulated cytokine generation in PLNC (**D**–**F**), ConA-stimulated cytokine production in MLNC (**G**–**I**), mRNA expression in PPC (**J**–**L**) were determined. Data are presented as mean +/− SD from 4 rats per group. *p < 0.05, Antb vs. Ctrl.

Spontaneous release of cytokines was measured in PLN cell culture supernatants. Cytokine levels are presented per whole PLN. There was more IFN-γ at 3 d.p.i., more IL-17 at 3 d.p.i. and 6 d.p.i. and more IL-10 at 6 d.p.i. in the PLN of antibiotic-treated rats (Fig. 6A-C). Production of cytokines was also measured in PLN cells re-stimulated with MBP *in vitro*. There was more IFN-γ and IL-17 produced at 3 d.p.i., while differences in IL-10 generation did not reach statistical significance (Fig. 6D–F). Cytokine generation was also measured in MLN stimulated with ConA, while cytokine mRNA expression was measured in PPC. RT-PCR was performed with PPC only, as we were not able to obtain enough PPC to generate cell culture supernatants in adequate quantities for ELISA. No difference in the level of the examined cytokines was observed between antibiotic-treated and control rats (Fig. 6G–L).

## Discussion

Gut microbiota and GALT have manifold interactions that have been shown to have an increasing significance in initiation and propagation, but also in regulation of CNS autoimmunity^4^. Accordingly, changes in gut microbiota composition in multiple sclerosis patients in comparison to healthy individuals have been observed^7–10^. Likewise, differences in gut microbiota composition and cellular composition of MLN and PP were observed in EAE-prone DA rats in comparison to EAE-resistant Albino Oxford rats^18,19^. Here, we present evidence that disturbance of gut microbiota by antibiotics worsens EAE in DA rats. Although the detrimental effects of antibiotics on chronic inflammatory disorders have been reported previously, e.g. in experimental psoriasis^20^, infection-induced Guillain Barré Syndrome in NOD mice^21^, diabetes in NOD mice^22^, and experimental systemic sclerosis^23^, numerous reports have revealed suppressive effects of antibiotic treatment in EAE^24–27^. Also, protective effects of antibiotics were reported in animal models of other neurological diseases, such as Parkinson’s disease^28^ and Alzheimer’s disease^29^, as well as of various chronic inflammatory diseases, such as rheumatoid arthritis^30^ and autoimmune uveitis^31^. The results of this study, as well as our recent finding that antibiotics provoke clinically-manifested EAE in otherwise EAE-resistant AO rats, show that antibiotic treatment can also be harmful in the case of CNS autoimmunity^32^. The contradiction between our results and those of the other groups could be explained by the fact that in the work of others adult animals and not pups were treated. A similar discrepancy was already reported in experimental psoriasis, as neonatal treatment of mice with antibiotics increased susceptibility to psoriasis, while it ameliorated the disease in adult animals^20^. Also, the difference in the kind of antibiotics applied has to be considered, as different antibiotics induce different variations in gut microbiota^33^. Accordingly, different antibiotics were reported to have dissimilar effects on autoimmunity. For instance, neomycin and vancomycin had different effects on gut microbiota and consequently opposite effects on diabetes in NOD mice^34^. Furthermore, DA rats were used in our study, while C57BL/6 mice were used in the previous EAE studies^24–27^. The subtypes of EAE induced in DA rats and C57BL/6 mice are different in many aspects, including antigens and adjuvants used in immunization, the course of the disease, and distribution of inflammatory infiltrates within the CNS^35^. Moreover, the gut microbiota of mice and rats are different from each other, as well from human gut microbiota. However, detailed comparative analysis of rat and mice gut microbiota show that the rat microbiota profile is more similar to the human microbiota^36^. Accordingly, germ-free rats were shown to be more prone to receive and retain human gut microbiota than mice^37^. These data imply an intriguing possibility that human gut microbiota would be affected by antibiotics in a similar way as rat gut microbiota. Importantly, germ-free mice are less susceptible to EAE induction^38^. Also, development of spontaneous EAE in transgenic mice is completely absent in germ-free mice^39^. To the best of our knowledge, EAE has not been investigated in germ-free rats, a line of investigation that is surely worth pursuing.

Strong dysbiosis caused by antibiotic treatment, both in terms of microbiota diversity and composition was observed in this study. Decreased microbiota diversity seems to be one of the most consistent findings in gut microbiota dysbiosis and has been repeatedly associated with the modern Western lifestyle and autoimmune diseases^40^. However, the long term effects of antibiotics on microbiota diversity have been only sporadically evaluated^41^. This study was designed to temporally separate intestinal microbiota dysbiosis from the induction of the CNS autoimmunity. Rats were receiving antibiotics in the first 4 weeks of their life, while induction of EAE was performed at 8 weeks of age. The effects of the antibiotic treatment on gut microbiota were evident at the time of weaning, but they had become discrete by the time of the immunization. This implies that intestinal microbiota dysbiosis occurring early in the lifetime, and not the direct effect of antibiotics or gut microbiota dysbiosis at the time of CNS autoimmunity initiation, was a contributing factor to EAE pathogenesis. Interestingly, the microbiota of antibiotic-treated rats and controls were not significantly different on day 0 post-induction, but the difference is present 12 days later when microbiota richness is less in antibiotic-treated rats. This result might imply that the disease itself impacts the composition of the microbiota. Recent microbiota studies have shown that misuse of antibiotics leads to transient or profound loss of the number of microbial species with eventual loss of microbial diversity and intestinal domination by pathogenic bacteria^42^. This effect is even more significant when it happens early in life, when the gut microbiota is not fully established^43^. On the other hand, many other factors could also influence gut microbiota composition and diversity. We assume that EAE induction could be one of the factors leading to perturbation of the gut microbiota, resulting in increased microbiota diversity in the control group. However, it might be assumed that the effect of antibiotics in the early life of antibiotic-treated rats had a permanent effect on the diversity of gut microbiota, leading to the inability of the rats to change their gut microbiota diversity after EAE induction. Increased diversity in the control group could be the protection mechanism by which the organism is trying to overcome the detrimental effects of EAE induction. This mechanism might be inefficient in antibiotic-treated rats due to the permanent loss of some important microbial groups, particularly *Firmicutes* and *Actinobacteria* being replaced by *Proteobacteria* and *Bacteroidetes* in antibiotic-treated animals.

Antibiotic-imposed gut microbiota dysbiosis occurring early in life could impact immune responses later in life. A balanced microbiota is imperative for maturation of a well-adjusted host immune system, including the proper regulation of Th1, Th2, Th17, and regulatory T cells^44^. The importance of proper microbial colonization for immune development was shown previously on germ-free animals, where the absence of gut microbiota caused impaired immune function^45^. Specifically, gut microbiota dysbiosis caused by the early life antibiotic treatment of chickens affected the specific antibody response and total IgM and IgY plasma concentrations even months after antibiotic treatment cessation, regardless of the recovery of gut microbiota composition. Importantly, the fecal microbiota of the antibiotic-treated chickens was restored and resembled that of the control animals two weeks after cessation of antibiotic treatment. The authors speculated that lack of lactobacilli and *Clostridia* (important for the maintenance of intestinal homeostasis, enhancement of the innate and adaptive immunity, regulation of the T cell- mediated immune responses, and attenuation of the inflammatory processes, together with inhibition of the pathogens’ growth) was responsible for the improper immune development and long-term T cell function impairment^45^. In parallel, the results of many clinical studies indicate an association between early life antibiotic treatment and the increased risk of developing autoimmune and metabolic diseases such as allergies, inflammatory bowel diseases, obesity, and neurological and brain dysfunction later in life^46^.

Further, it has recently been shown that gut microbiota dysbiosis during adolescence and young adulthood has a profound role in EAE pathogenesis in transgenic mice expressing the MS-associated HLA-DR2a gene and an MBP87-99/DR2a-specific 3A6 TCR gene isolated from an MS patient that spontaneously develop EAE^47^. Namely, it was shown that gut dysbiosis during adolescence and young adulthood can overcome immunological tolerance to MBP through the increase of complement C3 component and following reduction of Foxp3 and E3 ubiquitin ligase genes^47^. Similarly, we present evidence that Treg are limited in rats treated with antibiotics early in the lifetime.

It is plausible to assume that the mechanism behind the EAE-promoting effects of antibiotics observed in DA rats is the change in microbial diversity and composition, as well as consequent potentiation of Th1- and Th17-driven immune response and attenuation of Treg cells. Indeed, increases in IFN-γ and IL-17 production were observed in lymphoid tissues of rats treated with antibiotics. Also, decreases in Treg frequency and IL-10 production were observed in mesenteric lymph nodes and Peyer’s patches. However, Treg frequency and IL-10 levels were increased in antibiotic-treated rats in the lymph nodes draining the site of immunization 6 days post immunization. We have previously observed in various mouse strains that frequency of Treg rises in mesenteric lymph nodes but decreases in draining lymph nodes after immunization^48^. There, it was indicated that the observed discrepancy was a consequence of the different migratory properties of Treg cells regulated through expression of CCR2 and CCR6 and/or through the proliferative capacity of the cells. Thus, the observed discrepancy in Treg frequency in draining lymph nodes and gut-associated lymphoid tissue in the present study implies that proper migratory and proliferative regulation of Treg are disturbed after antibiotic treatment. This assumption is worthy of further investigation.

Importantly, the increased IFN-γ and IL-17 production was also detected within the CNS. This clearly indicates that inappropriate T cell regulation as a consequence of gut microbiota dysbiosis early in the lifetime of rats translates to increased CNS autoimmunity in EAE. Still, in future it would be important to assess the effects of the antibiotic treatment on EAE spinal cord histopathological data, including the type of cellular infiltrates, demyelination degree, microglia activation, and astrogliosis.

Despite the large inter-individual differences among the human population, it is becoming recognized that a “healthy” human gut microbiota is dominated by balanced proportions of *Bacteroidetes*, *Firmicutes*, *Actinobacteria*, *Proteobacteria* and *Verrucomicrobia*^49^. In contrast, the alteration of gut microbiota, called “dysbiosis”, has been reported in a number of diseases^50^. Regarding the gut microbiota composition in multiple sclerosis patients, the data have been rather inconsistent. According to Miyake *et al*.^8^, decreased abundance of *Firmicutes* (*Fecalibacterium*, *Anaerostipes*) and *Bacteroidetes* (*Prevotela*) was scored in the gut microbiota of patients with relapsing-remitting multiple sclerosis. On the other hand, Chen *et al*.^10^ showed decreased *Bacteroidetes* (*Parabacteroides* and *Prevotella*) and *Actinobacteria* (*Adlercreutzia* and *Collinsella*) and increased *Firmicutes* (*Lactobacillus* and *Coprobacillus*) and *Proteobacteria* (*Pseudomonas*, *Mycoplana* and *Haemophilus*), while Tremlett *et al*.^51^ reported only an increase in the abundance of *Actinobacteria* members in the faecal microbiome of pediatric patients with relapsing-remitting multiple sclerosis.

Moreover, the extensive use of antibiotics has been associated with microbiota dysbiosis and the increased risk of inflammatory and autoimmune diseases^52^. Interestingly, *Helicobacter pylori* was reported to have protective effects in patients suffering from multiple sclerosis, suppressing the induction of encephalitogenic Th1 and Th17 cells which lead to neuroinflammation^50^. In line with that, our results indicate that prolonged antibiotic treatment causes substantial gut microbiota disruption, followed by the decrease of major bacterial groups important for early gut colonization, such as lactobacilli and bifidobacteria. In particular, the results reveal significant replacement of *Firmicutes* (mainly *Clostridia* and *Bacilli*, including *Lactobacillaceae*) and *Actinobacteria* (particularly *Bifidobacteriaceae*) by *Proteobacteria* (predominantly γ*-Proteobacteria*, including *Enterobacteriaceae*) and *Bacteroidetes* in the early life stages of antibiotic-treated DA rats. The presence of *Bifidobacterium* and *Lactobacillus* has been generally appreciated as beneficial for the host, especially for the colonisation of infants’ gut^53^. On the other hand, *Proteobacteria*, particularly *Enterobacteriaceae*, were demonstrated previously as the major bacteria in neonatal mice, but those were supressed and replaced by *Bacteroidetes* and *Firmicutes* in adult mice^54^. Moreover, the presence of *Proteobacteria* in adult mice led to increased colonic inflammation^54^. Interestingly, at the genus level among the *Bacteroidete* phylum, increased abundance of *Bacteroides* and decreased abundance of CF231 (belonging to *Prevotelaceae* family) in the antibiotic-treated rats was revealed. However, one has to be aware that inconsistencies among the previously reported results regarding the abundance of various bacteria in multiple sclerosis patients could be related to the phylogenetic level of the changed bacterial groups determined in the studies, together with differences in methodology, sequencing techniques, the small size of cohorts, lack of longitudinal studies, confounding factors and the geographical origins of the patients^10^.

In addition, the results of this study reveal a decreased abundance of *Turicibacteriaceae* in antibiotic-treated rats. This is in accordance with our previous results indicating the potential role of *Turicibacter* sp. in prevention of EAE development and the alleviation of the disease’s symptoms^18^. Importantly, the presence of *Turicibacter* was positively correlated with the faecal concentration of butyric acid^55–58^. Butyric acid, as well as other SCFA, has been shown to have immunoregulatory effects^59,60^. The direct exposure of naïve CD4^+^ T lymphocytes to butyrate leads to their differentiation into Treg. Also, butyric acid contributes to the differentiation of Treg cells indirectly via tolerogenic activation of dendritic cells^60,61^. Particular members of gut microbiota participate in the fermentation of indigestible oligosaccharides, resulting in the production of SCFA^62^. In accordance with literature data^63^, the results of this study reveal a significant decrease in faecal SCFA concentration in antibiotic-treated DA rats compared to control groups. Interestingly, a significant increase of concentration of butyric acid, as well as the increase in the relative abundance of butyric acid in total SCFA, was observed in antibiotic-treated rats in comparison to control animals. In contrast, decreased concentrations of butyric acid were observed previously after oral ampicillin treatment^64^. However, in a study that determined an increase in SCFA production in lupus patients compared to healthy controls^65^, the authors assumed that gut microbiota dysbiosis contributed to the disease’s pathogenesis through metabolic alterations, as the absence of certain bacterial groups enabled colonization of other bacterial groups with a different metabolic pattern. Specifically, a reduced *Firmicutes*/*Bacteroides* ratio was related to increased SCFA fecal levels, particularly the level of propionate. Although butyrate is well known for its immunoregulatory properties, e.g. being able to attenuate bowel inflammation^59^, it was reported that the addition of butyrate to drinking water given to mice with dextran sulphate sodium-induced colitis aggravated the symptoms of the disease^66^. It was also shown that butyrate can cause the induction of Th17 response and the production of IL-23 in stimulated dendritic cells, exacerbating inflammation^66^. Moreover, recent results show that higher concentrations of butyric acid may stimulate the expression of transcription factor T-bet in T cells, thus inducing generation of IFN-γ-producing Tregs or conventional T cells^67^. Thus, the role of butyrate in mucosal immunity is not unequivocal. Also, increased levels of faecal content of butyric acid do not necessarily mean that this SCFA is available for gut cells. Indeed, the reduced expression of monocarboxylate transporter 1 (MTC 1) protein and the increased levels of butyric acid were observed in patients with inflammatory bowel disease^68,69^. The authors suggested that the increased concentrations of IFN-γ and TNF led to the negative regulation of expression of MTC 1, responsible for the transport of butyric acid. The results of our study indicate that the decreased production of SCFA by intestinal microbiota, together with potentially decreased transport of butyric acid to the host, could be, at least partially, responsible for the decrease in Treg abundance in the GALT and for consequent aggravation of EAE. Indeed, a lower proportion of Treg was observed in the mesenteric lymph nodes and Peyer’s patches of rats treated with antibiotics. Still, the exact role of SCFA, and butyrate in particular, in development and prognosis of autoimmune diseases, such as EAE and multiple sclerosis, requires further elucidation.

Our results suggest that gut microbiota disturbance in early childhood can predispose an individual towards CNS autoimmunity that can be provoked by independent stimuli at a later age. Although simultaneous application of different antibiotics in high doses and for an extremely long period is not likely to be used in humans, our results suggest caution in antibiotic use. Antibiotics are the most commonly used drugs in the paediatric populations of Western countries^17,70^. In fact, their consumption increased by 36% between 2000 and 2010^15^. Also, our data call upon epidemiological studies that would explore gut microbiota disturbances as a consequence of antibiotic treatment in the early childhood of multiple sclerosis patients. Actually, antibiotic-induced dysbiosis in children has already been proposed as a potential predisposing factor for various diseases, including inflammatory bowel disease, obesity and asthma^71,72^.

In conclusion, antibiotic-induced disturbance of gut microbiota in EAE-prone rats provokes more severe CNS autoimmunity. The effect is mediated through disturbance of Th/Treg balance. The results of this study contribute to the pool of knowledge regarding the complex relationship between gut microbiota and CNS autoimmunity.

## Methods

### EAE induction and evaluation

Female DA rats were bred and maintained in the local animal facility and their use was approved by the local ethics committee (Institute for Biological Research “Siniša Stanković”, N° 04-04/15) in accordance with Directive 2010/63/EU. Three to five rats were kept in the same cage. EAE was induced with rat spinal cord homogenate (SCH) in phosphate buffer saline (PBS, 50% w/v) mixed with an equal volume of complete Freund’s adjuvant (CFA, Difco, Detroit, MI) supplemented with 5 mg/ml of *M*. *tuberculosis* H37Ra (Difco). Alternatively, rats were immunized with myelin basic protein (guinea pig MBP, 50 μg/rat, kind gift from Professor Alexander Flügel, University of Göttingen, Germany), emulsified with an equal volume of CFA supplemented with 5 mg/ml of *M*. *tuberculosis* H37Ra. The animals were injected intradermally into hind limbs with 100 μL of either emulsion. The rats were monitored daily for clinical signs (c.s.) of EAE: 0, no clinical signs; 1, tail atony; 2, hind limb paresis; 3, hind limb paralysis; 4, moribund state or death. Intermediate scores were assigned if neurologic signs were of lower severity than typically observed. Cumulative c.s. was calculated as sum of daily c.s. Duration was number of days that the clinical signs were observed in each of the rats. Mean c.s. was calculated as cumulative c.s. divided by duration. In order to detect inflammatory infiltrates in the CNS of immunized animals, hematoxylin and eosin staining was performed. Five micrometres of transversal lumbosacral sections (L1–L5) were cut. Number of infiltrates and cells per infiltrate were counted from histological data obtained from 17 sections per group.

### Antibiotic treatment

Neosulfox (Sulfadimidine sodium 10% (w/w), neomycin sulfate 6%, oxytetracycline hydrochloride 4%, „Fm Pharm“ d.o.o., Subotica), 2.5 g/l Pentrexyl (ampicillin, Galenika, Belgrade, Serbia) were applied in drinking water and changed regularly every second day. The treatment of pregnant dams started two weeks before the delivery and was introduced to rats gradually from 0.5 g/l to 2.5 g/l. The full dosage was achieved one week after the beginning of the treatment. Rats were allowed to drink water with antibiotics *ad libitum* for additional 4 weeks *postpartum*. Then, rats were separated from mothers and were left without additional treatment till week 8 when they were immunized. Control rats were maintained in parallel. Breeding pairs for antibiotic treatment and controls were littermates.

### DGGE analysis, DNA sequencing and microbiome analyses

Faecal samples were collected both from antibiotic-treated and control animals, starting from dams at the time of delivery (14 days after the treatment with antibiotic was started) and from the offspring at the time of separation (28 days before the immunization, −28 d.p.i.), immunization (0 d.p.i.), EAE peak (12 d.p.i.) and recovery (31–35 d.p.i.), as given in Fig. 1A. All faecal samples were collected under clean and sterile conditions and immediately frozen at −20 °C. Genomic DNA was isolated using the standard protocol from the ZR Faecal DNA MiniPrep™ Kit (Zymo Research, Irvine, CA). About 100 to 150 mg of feces was taken from each sample vial under ongoing strict decontamination conditions (personnel using masks and gloves, and decontaminated clean sterile equipment in a sterile ventilated hood, Labconco Class II Type B2). All extracted DNA samples were stored at −20 °C. DNA was quantified by PicoGreen assay (Invitrogen, ThermoFisher Scientific, Waltham, MA USA). The quantities of obtained DNA from all specimens were sufficient for DGGE and 16S-based metagenomic sequencing. DGGE analysis and gel manipulation after electrophoresis was entirely performed as described previously^73^. The universal primer set complementary to 16S rRNA, specific for Eubacteria, U-968-GC-f pared with L1401-r, was used^74^. One sample from each antibiotic-treated and control group was used for 16S rRNA metagenomic sequencing, provided by Llúcia Martinez-Priego and the team at FISABIO Sequencing and Bioinformatics Service (via Science Exchange).

The processing of the DGGE profiles was done with PyElph software version 1.4. DGGE fingerprints were manually scored by the presence or absence of co-migrating bands, independent of intensity. Similarities between samples were determined by calculating similarity indices based on the Dice similarity coefficient: *Dsc* = *(2j/(a* + *b))* × *100* [%], where *j* is the number of bands common to samples A and B, and *a* and *b* are total number of band in samples A and B, respectively. Two identical profiles create a value of 100%, whereas two profiles without common bands result in a value of 0%.

### HPLC – UV analysis

Extraction of SCFA from faecal samples was performed as described previously^75^. Quantification of SCFA in faecal samples was performed by high- performance liquid chromatography (HPLC) using external calibration standards curves method. Four calibration standards were prepared at seven levels of concentration ranging from 0.5 mM to 50 mM for acetic acid (AA), propionic acid (PA), butyric acid (BA) and internal standard – succinic acid (SA). The calibration curves were constructed by plotting the relative peak area versus the molarity of the solution. Faecal concentrations of SCFA were calculated by using the following equation: SCFA (AA, PA, BA) = (organic acid in faecal sample × 6 × 10^−3^)/(succinic acid in faecal sample × mass of faecal sample) × 1000 [mmol/kg].

The HPLC – UV system consisted of LPG 3400SD HPLC pump, DAD 3000 detector, WPS 3000TSL auto sampler and TCC 3000SD column compartment, all from Thermo Scientific (Berda, The Netherlands). Chromatographic separation was performed on Hypersil Gold aQ column (150 mm × 4.6 mm) with particle size of 3 µm (Thermo Scientific, Breda, The Netherlands). The HPLC column was protected by a guard column of the same type. The column was thermostatized at 30 °C. The mobile phase consisted of 20 mM of NaH_2_PO_4_ in HPLC water (pH adjusted to 2.2 using phosphoric acid) [A] and 100% acetonitrile [B]. The UV detector was set at a wavelength of 210 nm. Data processing was performed using Chromeleon 6.8 software (Thermo Scientific).

### Isolation of cells, cell culturing and generation of supernatants

Cells of lymph nodes draining the site of immunization (popliteal lymph nodes, PLN) were obtained from rats immunized with an emulsion made of MBP and CFA. PLN cells (PLNC) were obtained by mechanical disruption. The isolation took place at 3 and 6 days post immunization (d.p.i.). Mesenteric lymph nodes (MLN) and Peyer’s patches (PP) were isolated from non-immunized rats and from rats on 0, 3, 6 d.p.i. For extraction of MLN and PP rats were immunized with an emulsion made of SCH and CFA. MLN cells (MLNC) and PP cells (PPC) were obtained by mechanical disruption. 5 × 10^6^/ml/well PLNC in 24-well plates (Sarstedt, Nümbrecht, Germany) were cultured in RPMI1640 medium (PAA Laboratories, Pasching, Austria) supplemented with 2% rat serum. The cells left un-treated for spontaneous release of cytokines or re-stimulated with MBP (10 µg/ml). 2.5 × 10^6^/ml MLNC were grown in RPMI1640 medium supplemented with 5% fetal calf serum (FCS, PAA Laboratories) and stimulated with concanavalin A (ConA, Sigma-Aldrich, 2.5 µg/ml). Cultures lasted for 24 h and subsequently cell culture supernatants were collected and kept frozen until assayed. Supernatants were also obtained from spinal cord homogenates. The whole spinal cord was homogenized in PBS (w/V at 1 g:2 ml) in a 2 ml Dounce All-Glass Tissue Grinder (DWK Life Sciences LLC, Rockwood, TN) by 5 strokes at large clearance and 5 strokes at small clearance. Spinal cord homogenate was centrifuged at 10000 g for 30 min and resulting supernatants were collected. For calculation of spontaneous cytokine level per PLN the following formula was applied: CxN/5, where C is level of a cytokine measured in cell culture supernatant and N is number of cells counted in a PLN (in millions).

### Cytofluorimetry

Cells were stained with the following antibodies: PE-conjugated anti-CD4 (OX35, eBioscience, San Diego, CA), FITC-conjugated anti-CD25 (NDS601, AbD Serotec, Oxford, UK), FoxP3 (FJK-16s, eBioscience). Appropriate isotype control antibodies were used where necessary to set gates for cell marker positivity. Analyses were performed on a CyFlow Space cytometer (Partec, Munster, Germany).

### ELISA

Cytokine concentration in cell culture supernatants was determined by sandwich ELISA using MaxiSorp plates (Nunc, Rochild, Denmark). For IL-10 detection Rat IL-10 DuoSet ELISA was used (R&D Systems, Minneapolis, MN). For IFN-γ and IL-17 detection the following eBioscience antibodies were used: anti-rat IFN-γ (DB1), anti-rat IFN-γ biotinylated rabbit polyclonal, anti-mouse/rat IL-17A (eBio17CK15A5) and anti-mouse/rat IL-17A biotinylated (eBio17B7). Samples were analyzed in duplicates and the results were calculated using standard curves made on the basis of known concentrations of the recombinant rat IL-10 (R&D Systems), IFN-γ and IL-17 (Peprotech, Rocky Hill, NJ).

### Statistical analysis

Two-way ANOVA followed with Student’s t test (two-tailed) or Tukey’s multiple comparison test, as appropriate was performed for statistical analysis. A p value less than 0.05 was considered statistically significant. Area under curve (AUC) was calculated by a numerical integration method based on the trapezoidal rule. Raw 16S rRNA sequences were processed in QIIME^76,77^ with default settings, except for taxonomic annotation which was done as suggested previously^78^. Raw data are submitted to European Nucleotide Archive (ENA) (https://www.ebi.ac.uk/ena) under accession number PRJEB26086, secondary accession number ERP108059.

OTU picking and taxonomic annotation of clustered sequences was performed in QIIME against both GreenGenes database and HIT.db^78^. All subsequent statistical analysis was done in R^79^. Shannon index was calculated as a measure of alpha diversity, beta diversity was assessed using weighted unifrac as a distance measure, and RDA analysis were all performed in R 3.4.3^79^ using packages ape^80^ and phyloseq^81^. Differential expression at family and genus level was assessed using ALDEx2 package^82^ under compositional data analysis paradigm. P values were adjusted with Benjamini-Hochberg transformation.

### Ethics approval and consent to participate

Animal experiments were approved by the local ethics committee (Institute for Biological Research “Siniša Stanković”, N° 04-04/15).

## Supplementary information

Dataset 1
