# Supplementary material for: Oral neonatal antibiotic treatment perturbs gut microbiota and aggravates central nervous system autoimmunity in Dark Agouti rats
Source: Sci Rep. 2019 Jan 29;9:918. doi: 10.1038/s41598-018-37505-7 (PMC6351648; doi:10.1038/s41598-018-37505-7)
Supplement: Supplementary file 1 — Dataset 1 [file 41598_2018_37505_MOESM1_ESM.pdf]

Oral neonatal antibiotic treatment perturbs gut microbiota and aggravates central nervous system autoimmunity in Dark Agouti rats

Suzana Stanisavljević[1], Aleksa Čepić[2], Svetlana Bojić[3], Katarina Veljović[2], Sanja Mihajlović[2], Neda Đedović[1], Bojan Jevtić[1], Miljana Momčilović[1], Milica Lazarević[1], Marija Mostarica Stojković[4], Đorđe Miljković[1], Nataša Golić[2*]

*Corresponding author

Nataša Golić, Laboratory for Molecular Microbiology, Institute of Molecular Genetics and Genetic Engineering, University of Belgrade, Vojvode Stepe 444a, Belgrade, Serbia

Correspondence to natasag@imgge.bg.ac.rs

Affiliations:

[1]Department of Immunology, Institute for Biological Research "Siniša Stanković", University of Belgrade, Serbia

[2]Laboratory for Molecular Microbiology, Institute of Molecular Genetics and Genetic Engineering, University of Belgrade, Serbia

[3]HITTest doo, Belgrade, Serbia

[4]Institute for Microbiology and Immunology, School of Medicine, University of Belgrade, Serbia

MurineMS

First we load required libraries

```
library(phyloseq)
library(phytools)
```

```
## Loading required package: ape
```

```
## Loading required package: maps
```

```
library(ggplot2)
library(dendextend)
```

```
##
## ---------------------
## Welcome to dendextend version 1.7.0
## Type citation('dendextend') for how to cite the package.
##
## Type browseVignettes(package = 'dendextend') for the package vignette.
## The github page is: https://github.com/talgalili/dendextend/
##
## Suggestions and bug-reports can be submitted at: https://github.com/talgalili/dendextend/i
ssues
## Or contact: <tal.galili@gmail.com>
##
##  To suppress this message use:  suppressPackageStartupMessages(library(dendextend))
## ---------------------
```

```
##
## Attaching package: 'dendextend'
```

```
## The following object is masked from 'package:phytools':
##
##     untangle
```

```
## The following objects are masked from 'package:ape':
##
##     ladderize, rotate
```

```
## The following object is masked from 'package:stats':
##
##     cutree
```

```
library(zCompositions)
```

```
## Loading required package: MASS
```

```
## Loading required package: NADA
```

```
## Loading required package: survival
```

```
##
## Attaching package: 'NADA'
```

```
## The following object is masked from 'package:stats':
##
##     cor
```

```
## Loading required package: truncnorm
```

```
library(compositions)
```

```
## Loading required package: tensorA
```

```
##
## Attaching package: 'tensorA'
```

```
## The following object is masked from 'package:base':
##
##     norm
```

```
## Loading required package: robustbase
```

```
##
## Attaching package: 'robustbase'
```

```
## The following object is masked from 'package:survival':
##
##     heart
```

```
## Loading required package: energy
```

```
## Loading required package: bayesm
```

```
## Welcome to compositions, a package for compositional data analysis.
## Find an intro with "? compositions"
```

```
##
## Attaching package: 'compositions'
```

```
## The following object is masked from 'package:NADA':
##
##     cor
```

```
## The following object is masked from 'package:ape':
##
##     balance
```

```
## The following objects are masked from 'package:stats':
##
##     cor, cov, dist, var
```

```
## The following objects are masked from 'package:base':
##
##     %*%, scale, scale.default
```

```
library(vegan)
```

```
## Loading required package: permute
```

```
##
## Attaching package: 'permute'
```

```
## The following object is masked from 'package:dendextend':
##
##     shuffle
```

```
## Loading required package: lattice
```

```
## This is vegan 2.4-6
```

```
library(ALDEx2)
```

Then we separate otu table and taxonomy table that came together as a result of Qiime processing; otu_table is included in the repository, as well as sample metadata and tree

```
otu_table_HIT <- read.csv("data/otu_table_HIT.csv")
tax_raw_HIT <- otu_table_HIT[,12]
rownames(otu_table_HIT)<- otu_table_HIT[,1]
###
tax_rat_HIT <- data.frame(OTU=rownames(otu_table_HIT), p=NA, c=NA, o=NA, f=NA, g=NA, s=NA)
for (i in 1:length(tax_raw_HIT)){
  tax_rat_HIT$p[i]<- unlist(strsplit(as.character(tax_raw_HIT[i]), ";"))[1]
```

```
  tax_rat_HIT$c[i]<- unlist(strsplit(as.character(tax_raw_HIT[i]), ";"))[2]
  tax_rat_HIT$o[i]<- unlist(strsplit(as.character(tax_raw_HIT[i]), ";"))[3]
  tax_rat_HIT$f[i]<- unlist(strsplit(as.character(tax_raw_HIT[i]), ";"))[4]
  tax_rat_HIT$g[i]<- unlist(strsplit(as.character(tax_raw_HIT[i]), ";"))[5]
  tax_rat_HIT$s[i]<- unlist(strsplit(as.character(tax_raw_HIT[i]), ";"))[6]
}
row.names(tax_rat_HIT)<- tax_rat_HIT$OTU
tax_rat_HIT <- tax_rat_HIT[,-1]
tax_rat_HIT <- tax_table(tax_rat_HIT)
```

```
## Warning in .local(object): Coercing from data.frame class to character matrix
## prior to building taxonomyTable.
## This could introduce artifacts.
## Check your taxonomyTable, or coerce to matrix manually.
```

```
#
row.names(tax_rat_HIT) <- rownames(otu_table_HIT)
#
otu_rat_HIT <- otu_table(otu_table_HIT[,-c(1,12)], taxa_are_rows=TRUE)
head(otu_rat_HIT)
```

```
## OTU Table:          [6 taxa and 10 samples]
##                      taxa are rows
##                                 M9 M4  M8 M6 M7 M3 M2  M10 M1 M5
## Prevotella_bryantii_AF396925     2  0   0  0  0  0  0    0  0  0
## Actinomyces_georgiae_GU561319    0  2   1  0  0  0  0    0  0  0
## Gemella_haemolysans_NR_025903.1 39  0   0  5  4 12  3    0  0  0
## Filifactor_alocis_JN713151       1  0   0  0  0  0  0    0  0  0
## OTU1240                          5  1   0 10 17 25 16    0  0  0
## Citrobacter_braakii_JN644584     0 72 625  0  0  0  1 1902  2  6
```

combining all into the phyloseq object

```
phy<-read.newick("data/HITdb.tre")
phy<-collapse.singles(phy)
phy<-multi2di(phy)
treRatHIT <-phy
#tree imported
if (min(phy$edge.length) == 0) { phy$edge.length = phy$edge.length + 0.00001 }
###### now sample data
qiimeMap <- read.delim("data/qiimeMap.txt")
head(qiimeMap)
```

```
##   SampleID BarcodeSequence LinkerPrimerSequence InputFileName Description
## 1       A1              NA                   NA      A1_1.fna       human
## 2       A2              NA                   NA      A2_1.fna       human
## 3       A4              NA                   NA      A4_1.fna       human
## 4       A5              NA                   NA      A5_1.fna       human
## 5       A6              NA                   NA      A6_1.fna       human
## 6       A7              NA                   NA      A7_1.fna       human
##   AntibioticTreatment ChildTime
```

```
## 1                <NA>      <NA>
## 2                <NA>      <NA>
## 3                <NA>      <NA>
## 4                <NA>      <NA>
## 5                <NA>      <NA>
## 6                <NA>      <NA>
```

```
qiimeRat <- qiimeMap[qiimeMap$Description=="rat",]
rownames(qiimeRat) <- qiimeRat$SampleID
sampleRat <- sample_data(qiimeRat)
#######
phyloRatHIT <- phyloseq(otu_rat_HIT, sampleRat, tax_rat_HIT, treRatHIT)
summary(phyloRatHIT)
```

```
##   Length    Class     Mode
##        1 phyloseq       S4
```

```
class(phyloRatHIT)
```

```
## [1] "phyloseq"
## attr(,"package")
## [1] "phyloseq"
```

```
phyloRatHIT
```

```
## phyloseq-class experiment-level object
## otu_table()   OTU Table:         [ 1145 taxa and 10 samples ]
## sample_data() Sample Data:       [ 10 samples by 7 sample variables ]
## tax_table()   Taxonomy Table:    [ 1145 taxa by 6 taxonomic ranks ]
## phy_tree()    Phylogenetic Tree: [ 1145 tips and 1144 internal nodes ]
```

more metadata

```
sample_data(phyloRatHIT)$AntibioticTreatment <- c("antibiotic-free","antibiotic","antibiotic",
"antibiotic-free","antibiotic-free","antibiotic-free","antibiotic-free","antibiotic","antibiot
ic","antibiotic")
sample_data(phyloRatHIT)$ChildTime <- c("-28.dpi","12.dpi","dams","31-35.dpi","dams", "12.dpi"
,"0.dpi","-28dpi","0.dpi","31-35.dpi")
sample_data(phyloRatHIT)$ChildTime <- factor(sample_data(phyloRatHIT)$ChildTime, levels=c("dam
s", "-28.dpi", "0.dpi", "12.dpi", "31-35.dpi"))
```

b&rarefying and plotting alpha diversity measure

```
set.seed(123)
alpha_meas = c("Shannon")
phyloRatHITrar <- rarefy_even_depth(phyloRatHIT)
```

```
## You set `rngseed` to FALSE. Make sure you've set & recorded
##  the random seed of your session for reproducibility.
```

```
## See `?set.seed`
```

```
## ...
```

```
## 234OTUs were removed because they are no longer
## present in any sample after random subsampling
```

```
## ...
```

```
sample_data(phyloRatHITrar)$time_point<-
ifelse(sample_data(phyloRatHITrar)$ChildTime=="razdvoj", "-28 dpi",
                                   ifelse(sample_data(phyloRatHITrar)$ChildTime=="12dpi", "12
dpi",
                                                          ifelse(sample_data(phyloRatHITrar)$Ch
ildTime=="mother", "dams",
                                                                 ifelse(sample_data(phyloRatHIT
rar)$ChildTime=="3135dpi", "31-35 dpi",
                                                                        ifelse(sample_data(phy
loRatHITrar)$ChildTime=="3135dpi", "31-35 dpi",
                                                                               ifelse(sample_d
ata(phyloRatHITrar)$ChildTime=="0dpi", "0 dpi", NA))))))
p <- plot_richness(phyloRatHITrar,"time_point","AntibioticTreatment", measures=alpha_meas)
#some level ordering
sample_data(phyloRatHITrar)$time_point <- factor(sample_data(phyloRatHITrar)$time_point, level
s=c("dams", "-28 dpi","0 dpi","12 dpi", "31-35 dpi"))

ggplot(p$data, aes(x=AntibioticTreatment, y=value, color=samples)) +geom_point(size=3)+geom_bo
xplot(data=p$data, aes(x=AntibioticTreatment, y=value, color=NULL), alpha=0.1)+theme_bw()+labs
(x="", y="Shannon index")+theme(axis.text.x=element_text(size=18), axis.title.y=element_text(s
ize=18))
```

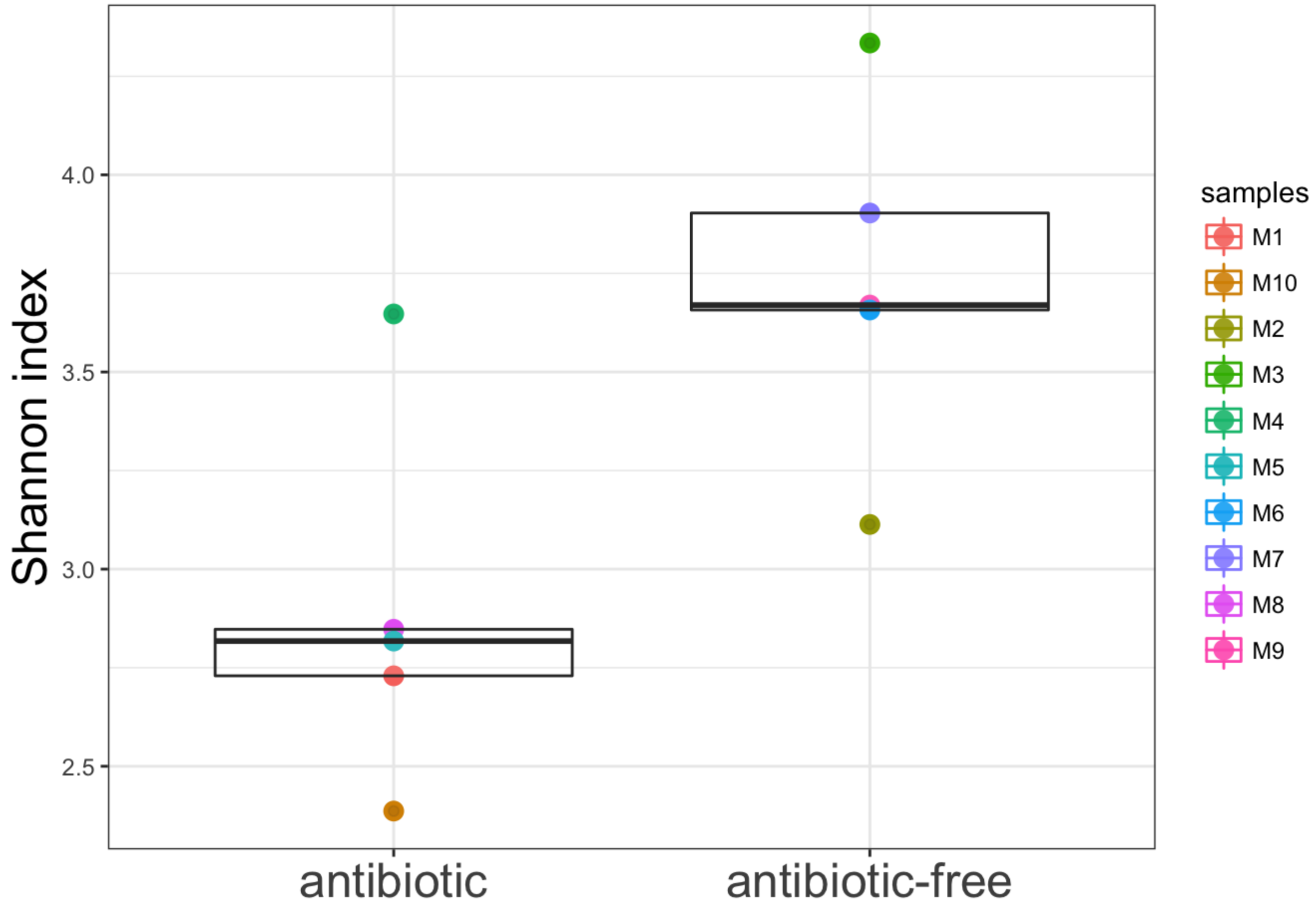

Next we do some ordination (unconstrained)

```
uniRatHIT <- distance(phyloRatHITrar, "wunifrac")
#
PCoA.ratHIT <- ordinate(phyloRatHITrar, method="PCoA", distance=uniRatHIT)
plot_scree(PCoA.ratHIT, "Scree plot for Rat data, UniFrac/PCoA")
```

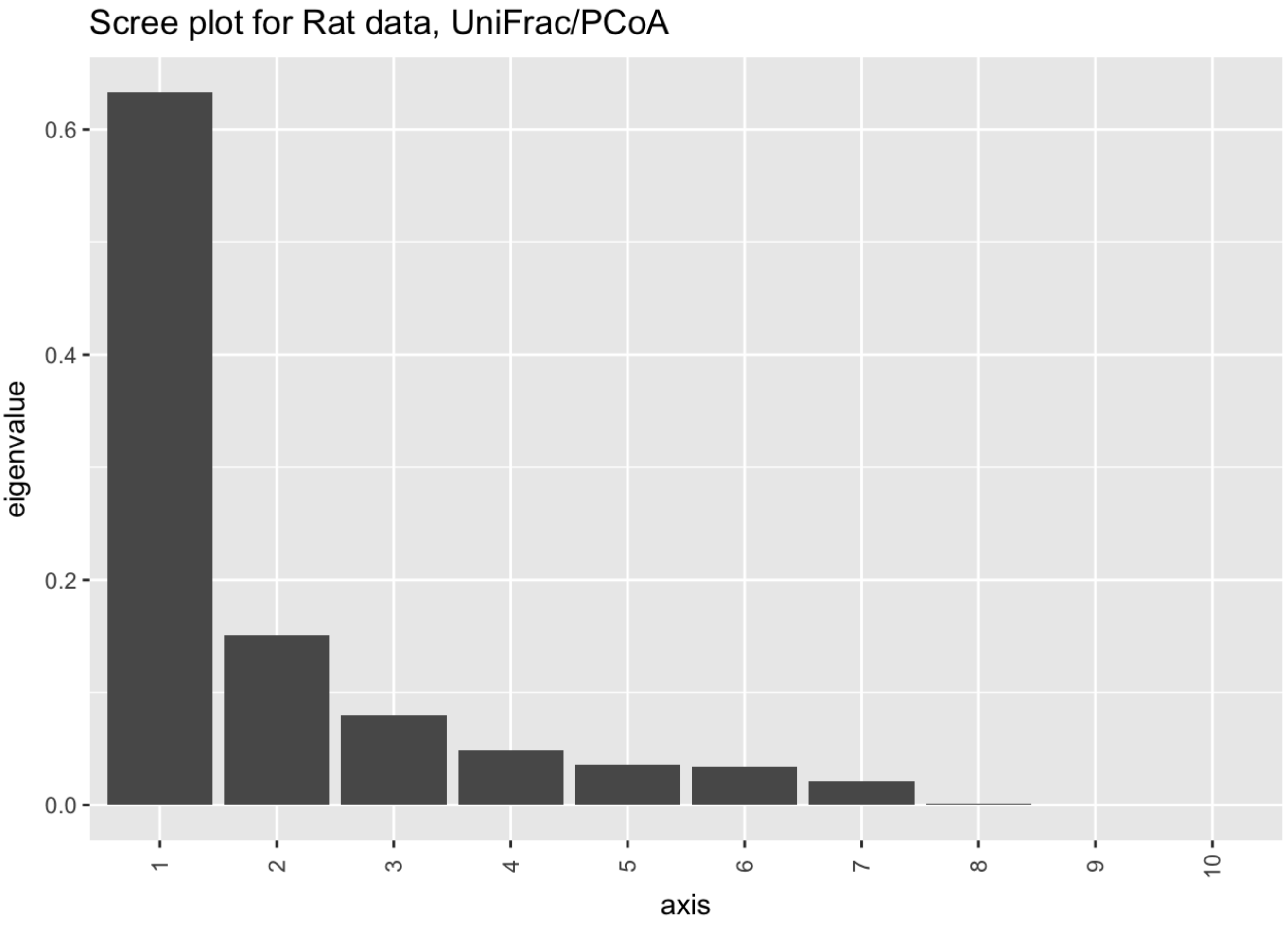


```
p12 <-plot_ordination(phyloRatHITrar, PCoA.ratHIT, axes=c(1, 2),"SampleID", color="AntibioticT
reatment")+geom_point(size=5) + geom_path() + scale_colour_hue(guide = FALSE)
p12
```

Now we use

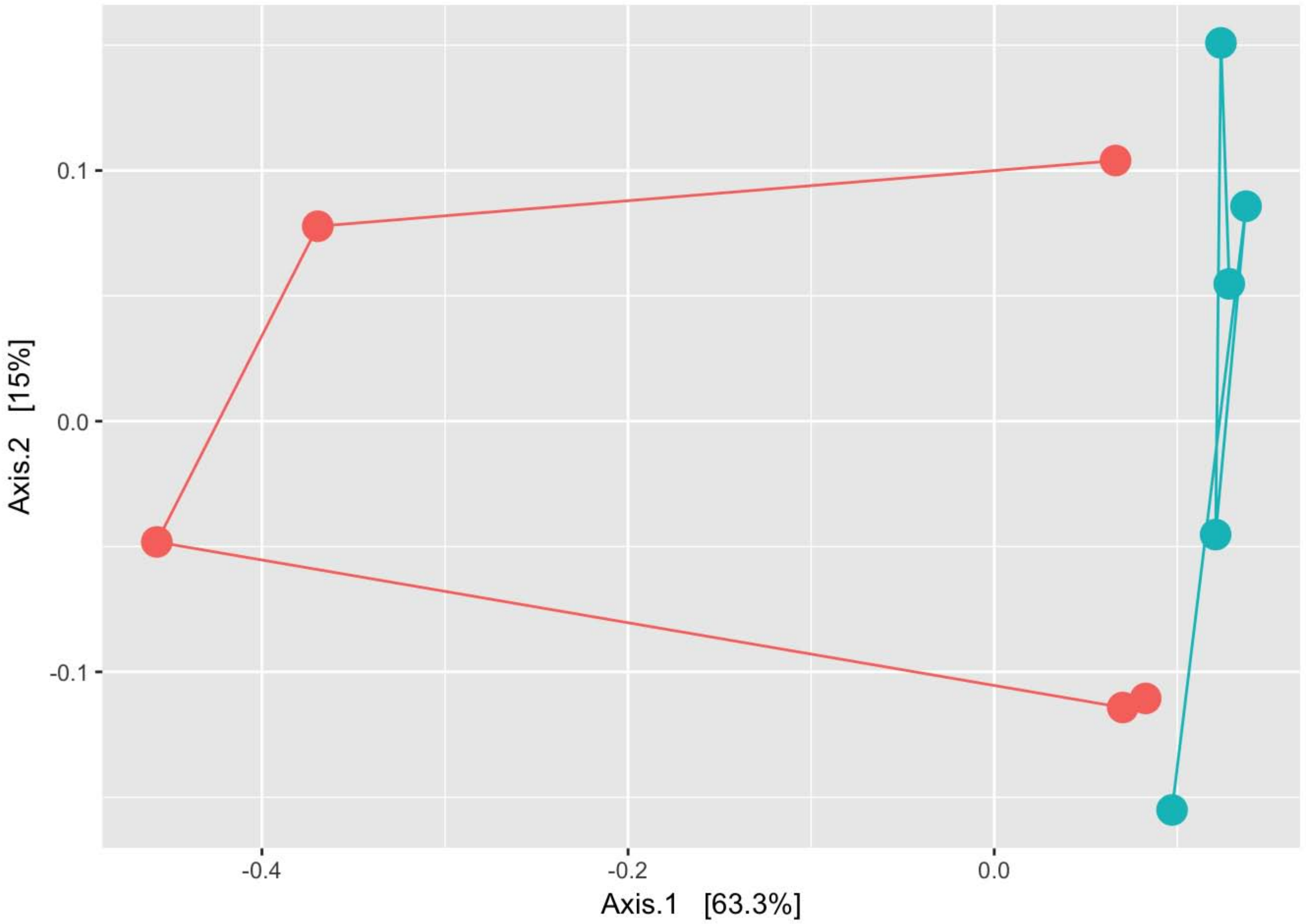

the same weighted unifrac distance to do the clustering

```
rat.hclustHIT<-hclust(d=uniRatHIT,method="ward.D")
rat.dendHIT <- as.dendrogram(rat.hclustHIT)
###some label manipulation
labels(rat.dendHIT)
```

```
##  [1] "M8"  "M10" "M1"  "M5"  "M9"  "M7"  "M6"  "M2"  "M4"  "M3"
```

```
data.clust <- data.frame(sampleID = c("M8","M10","M1","M5","M9","M7","M6","M2","M4","M3"))
data.clust$AntibioticTreatement <- sample_data(phyloRatHITrar)[as.character(data.clust$sampleID
),"AntibioticTreatment"]
data.clust$AntibCol <- ifelse(data.clust$AntibioticTreatment=="antibiotic-free", "deepskyblue2
", "red1")
data.clust$ChildTime <- sample_data(phyloRatHITrar)[as.character(data.clust$sampleID),"ChildTi
me"]
#labels(rat.dendHIT) <- data.clust$ChildTime
labels(rat.dendHIT) <- c("dams", "-28.dpi", "0.dpi", "  31-35.dpi","-28.dpi", "dams","31-35.d
pi", "0.dpi","12.dpi", "12.dpi")
labels_colors(rat.dendHIT)<- data.clust$AntibCol
plot(rat.dendHIT)
```

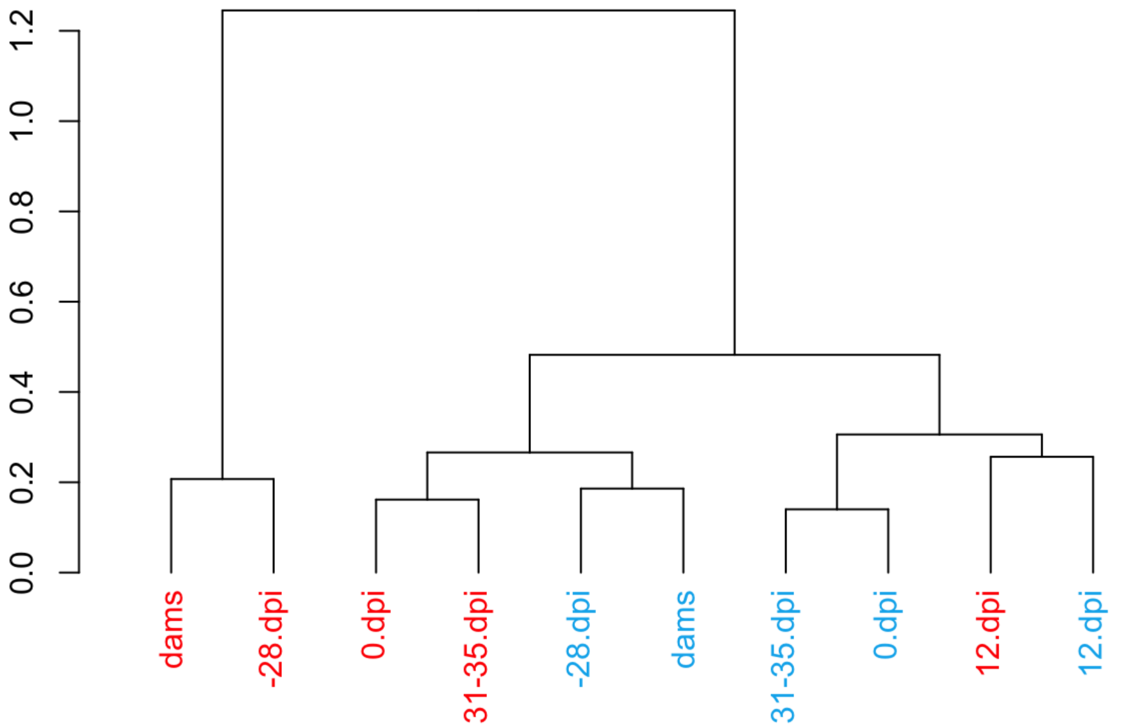


```
#this is the code for hierarchical clustering tree from the paper
```

now exploratory phylogenetic tree

```
RatHIT.imun <- subset_taxa(phyloRatHITrar, ta5 %in% c(" Peptococcus", " Peptoclostridium"," P
arasutterella", " Collinsella", " Turicibacter"))
plot_tree(RatHIT.imun, color="ChildTime", size="Abundance",shape="AntibioticTreatment", nodela
bf=nodeplotblank, ladderize="left", base.spacing=0.04,label.tips="ta6" )
```

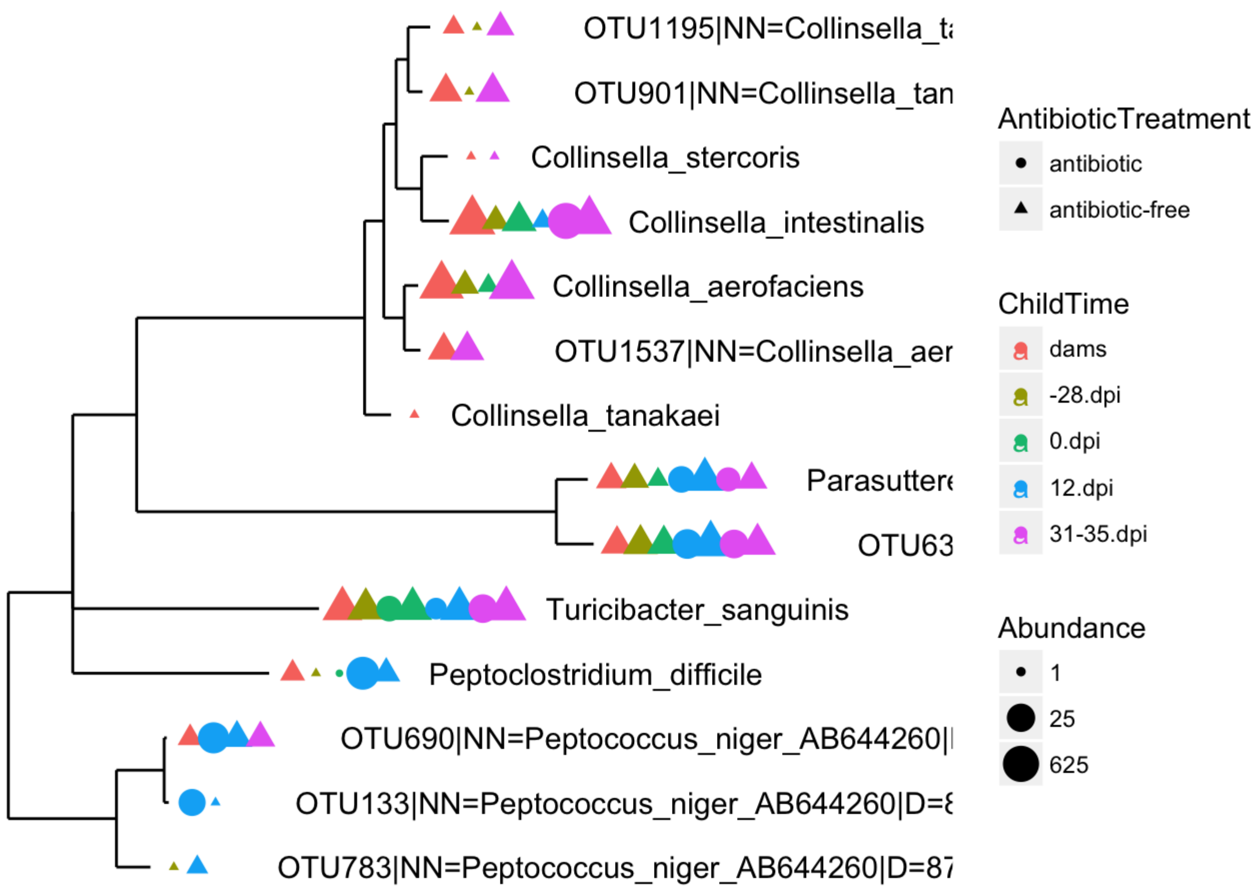


```
#this is the part of phylogenetic tree, from the paper
```

Now we are entering the realm of compositional data analysis framework

```
d.pro.HIT <- cmultRepl(t(otu_table(phyloRatHIT)), method="CZM", output="counts")
```

```
## No. corrected values:  186
```

```
# accounting  for zero counts
d.pro.HIT <- t(d.pro.HIT)
d.pro.HIT <- as.data.frame(d.pro.HIT)
d.pro.HIT$genus <- tax_table(phyloRatHIT)[,"ta5"]
#now aggregate, and do the clr transformation
genusRatHIT <-aggregate(.~genus, data=d.pro.HIT[,1:11], sum)
d.clr.abund.HIT <- t(apply(genusRatHIT[,-1], 2, function(x){log(x) - mean(log(x))}))
colnames(d.clr.abund.HIT) <- genusRatHIT[,1]
```

constrained ordination, by antibiotic usage

```
rda.part <- rda(d.clr.abund.HIT~AntibioticTreatment, data=data.frame(sample_data(phyloRatHIT))
)
##
scor <- scores(rda.part, display=c("sp", "cn", "bp", "wa"), scaling=2)
##
```

```
numeric_centroids <- data.frame(scor$centroids)
numeric_centroids$AntibioticTreatment <- c("antibiotic", "antibiotic-free")
#ggplot(numeric_centroids, aes(x=RDA1, y=PC1))+geom_text(aes(label=AntibioticTreatment))
##
species_centroids <- data.frame(scor$species)
species_centroids$species_names <- rownames(species_centroids)
##introducing criteria to avoid clutter in the middle of the plot
species_centroids$kriterijum <- sqrt(species_centroids$RDA1^2+species_centroids$PC1^2)
#
index.spec <- species_centroids$kriterijum > 0.75
species_centroids.over <- species_centroids[index.spec,]
#
site_scores <- data.frame(scor$sites)
site_scores$ChildTime <- sample_data(phyloRatHIT)$ChildTime
site_scores$SampleID <- sample_data(phyloRatHIT)$SampleID
site_scores$AntibioticTreatment<- sample_data(phyloRatHIT)$AntibioticTreatment
#
df_ell.antib<- data.frame()
veganCovEllipse <- function(cov, center=c(0,0), scale=1, npoints=100){
  theta <- (0:npoints)*2*pi/npoints
  Circle <- cbind(cos(theta), sin(theta))
  t(center+scale*t(Circle%*%chol(cov)))
}
site_scores$AntibioticTreatment <- factor(site_scores$AntibioticTreatment)
for (g in levels(site_scores$AntibioticTreatment)){
  df_ell.antib<- rbind(df_ell.antib,cbind(as.data.frame(with(site_scores[site_scores$Antibioti
cTreatment==g,], veganCovEllipse(cov.wt(cbind(RDA1,PC1),wt=rep(1/length(RDA1), length(RDA1)))$
cov, center=c(mean(RDA1), mean(PC1))))),AntibioticTreatment=g))}
```

```
## Warning in structure(c(), class = c(class(x), class(y))): Calling 'structure(NULL, *)' is
deprecated, as NULL cannot have attributes.
##   Consider 'structure(list(), *)' instead.

## Warning in structure(c(), class = c(class(x), class(y))): Calling 'structure(NULL, *)' is
deprecated, as NULL cannot have attributes.
##   Consider 'structure(list(), *)' instead.
```

```
#
RDA.plot.genus <-ggplot(site_scores, aes(x=RDA1, y=PC1))+geom_point(aes(colour=AntibioticTreat
ment, shape=AntibioticTreatment), size=5)+geom_path(data=df_ell.antib, aes(x=RDA1, y=PC1, grou
p=AntibioticTreatment, colour=AntibioticTreatment), alpha=0.3, linetype=1)+
  geom_segment(data=species_centroids.over, aes(x=0, xend=2*RDA1, y=0, yend=8.5*PC1), arrow=ar
row(length=unit(0.25, "cm")), colour="grey4", alpha=0.4, linetype=2)+
  geom_text(data=species_centroids.over, aes(x=2.5*RDA1, y=9.3*PC1,label=species_names), size=
4, colour="black")+theme_bw()
RDA.plot.genus
```

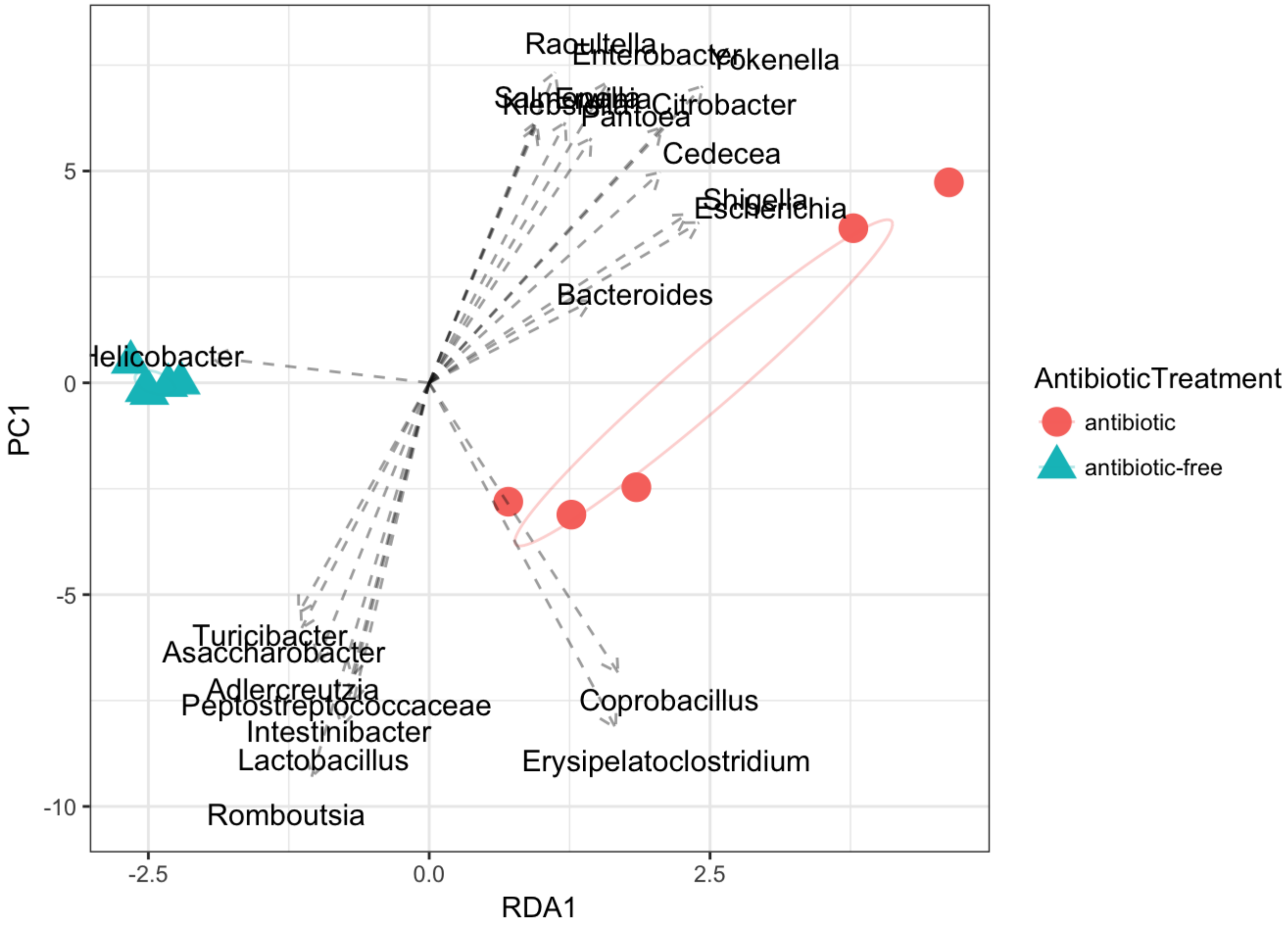


```
#this is the RDA plot from paper
#### now formal testing
anova(rda.part, by="terms")
```

```
## Permutation test for rda under reduced model
## Terms added sequentially (first to last)
## Permutation: free
## Number of permutations: 999
##
## Model: rda(formula = d.clr.abund.HIT ~ AntibioticTreatment, data = data.frame(sample_data(
phyloRatHIT)))
##                      Df Variance      F Pr(>F)
## AntibioticTreatment  1   149.88 4.8738  0.007 **
## Residual             8   246.01
## ---
## Signif. codes:  0 '***' 0.001 '**' 0.01 '*' 0.05 '.' 0.1 ' ' 1
```

and now testing with ALDEx

```
d.aldex <- data.frame(otu_table(phyloRatHITrar))
d.aldex$genus <- tax_table(phyloRatHITrar)[,"ta5"]
index.NA.genus <- !is.na(d.aldex$genus)
#removing taxa not annotated on genus level
d.genus <- d.aldex[index.NA.genus,]
genusRatHIT <-aggregate(.~genus, data=d.genus[,1:11], sum)
```

```
rownames(genusRatHIT) <- genusRatHIT$genus
##
conds.aldex <- c("antibiotic-free","antibiotic","antibiotic-free","antibiotic","antibiotic-free","antibiotic-free","antibiotic-free","antibiotic","antibiotic","antibiotic")
x <- aldex.clr(genusRatHIT[,2:11], conds.aldex,mc.samples=128, verbose=TRUE)
```

```
## [1] "operating in serial mode"
## [1] "removed rows with sums equal to zero"
## [1] "computing center with all features"
## [1] "data format is OK"
## [1] "dirichlet samples complete"
## [1] "clr transformation complete"
```

```
#calculating p value for each replicate
x.t <- aldex.ttest(x, conds.aldex)
```

```
## [1] "running tests for each MC instance:"
## |------------(25%)----------(50%)----------(75%)----------|
```

```
x.e <- aldex.effect(x, conds.aldex, verbose=TRUE)
```

```
## [1] "operating in serial mode"
## [1] "sanity check complete"
## [1] "rab.all  complete"
## [1] "rab.win  complete"
## [1] "rab of samples complete"
## [1] "within sample difference calculated"
## [1] "between group difference calculated"
## [1] "group summaries calculated"
## [1] "effect size calculated"
## [1] "summarizing output"
```

```
x.all <- data.frame(x.e, x.t)
sig <- which(x.all$wi.eBH <=0.05)
sig
```

```
## integer(0)
```

..no genus was found significantly associated with antibiotic usage

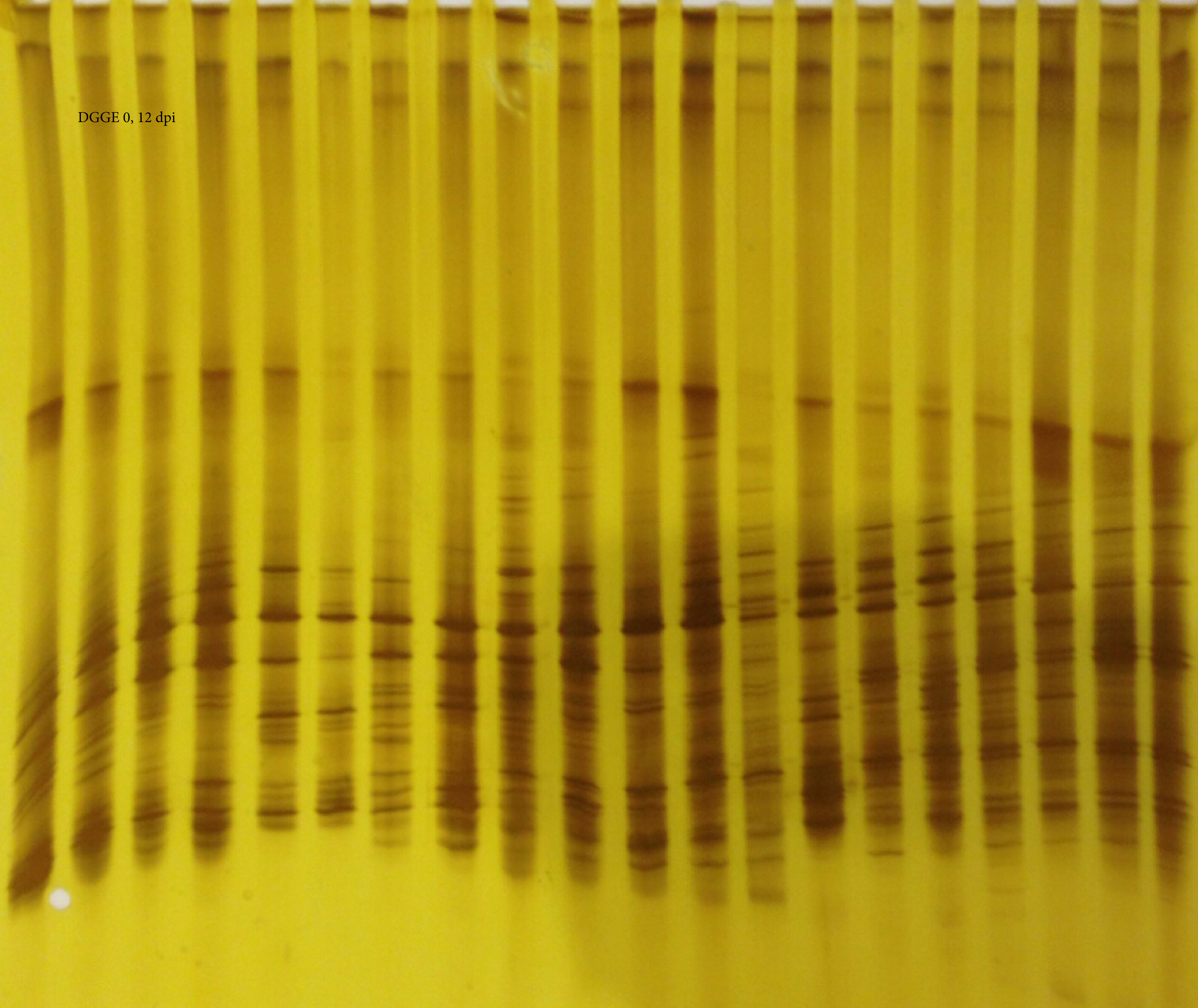
DGGE 0, 12 dpi

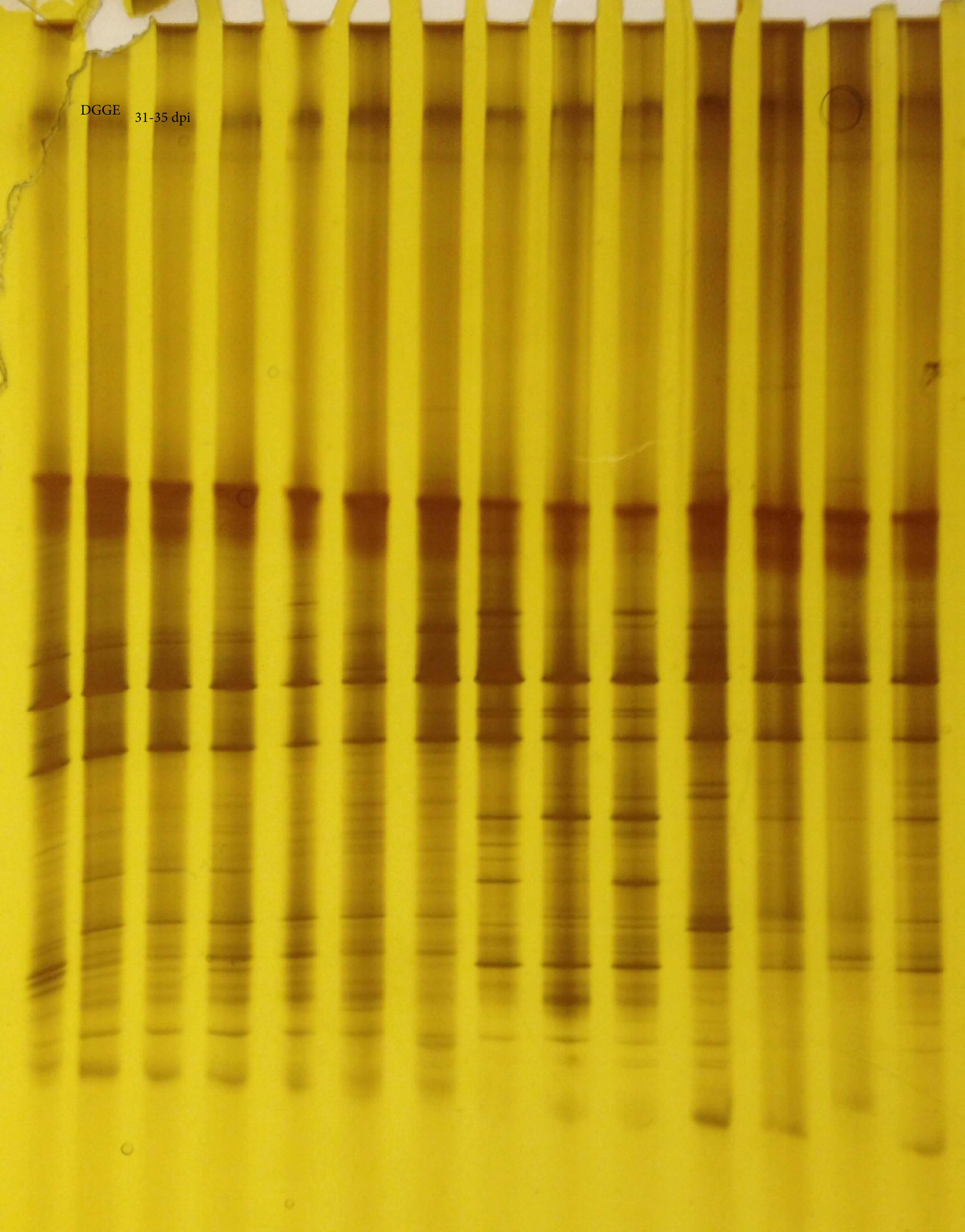
DGGE
31-35 dpi

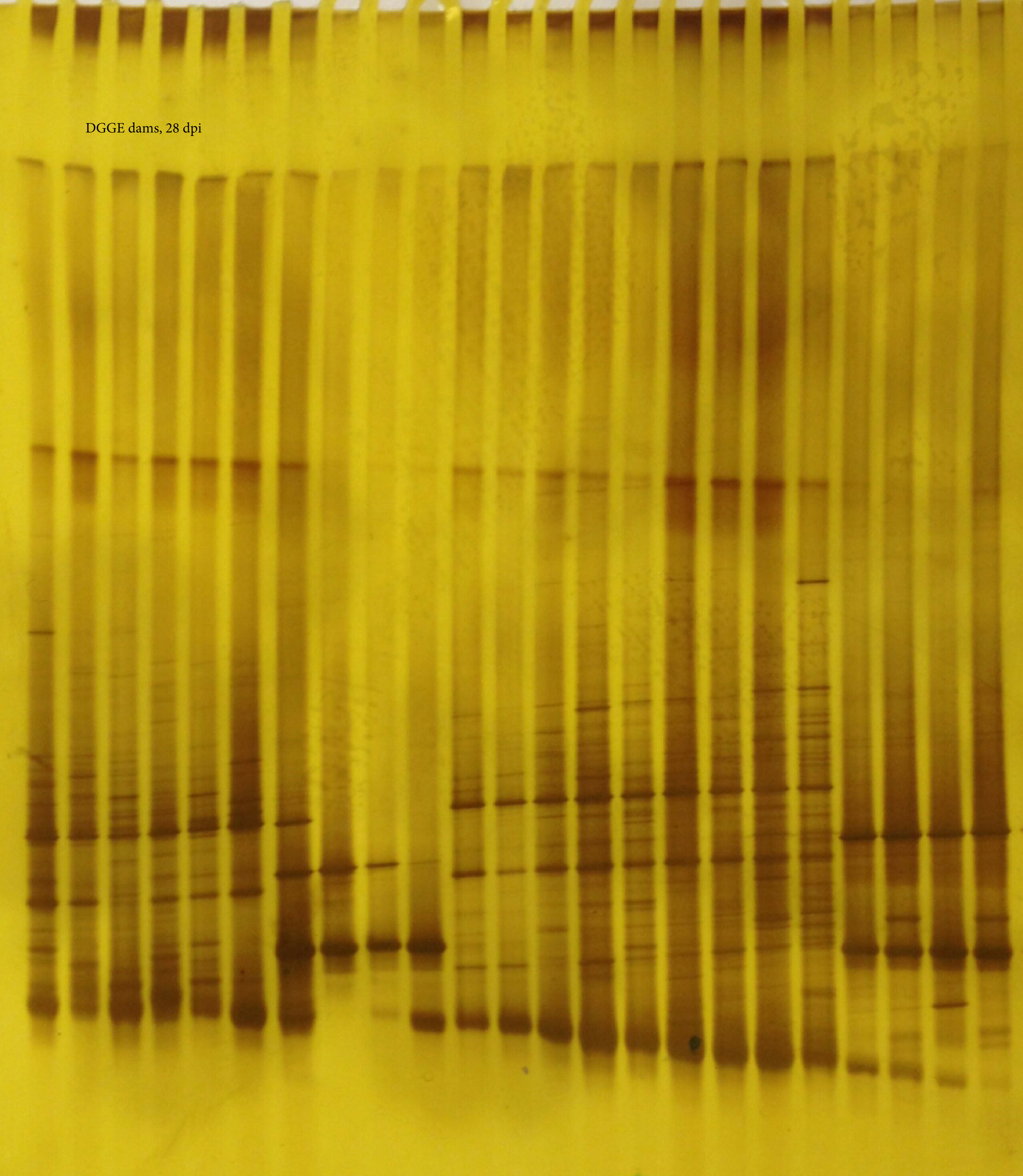
DGGE dams, 28 dpi
